# Crystal structure of a bacterial photoactivated adenylate cyclase determined by serial femtosecond and serial synchrotron crystallography

**DOI:** 10.1107/S2052252524010170

**Published:** 2024-10-29

**Authors:** Sofia M. Kapetanaki, Nicolas Coquelle, David von Stetten, Martin Byrdin, Ronald Rios-Santacruz, Richard Bean, Johan Bielecki, Mohamed Boudjelida, Zsuzsana Fekete, Geoffrey W. Grime, Huijong Han, Caitlin Hatton, Sravya Kantamneni, Konstantin Kharitonov, Chan Kim, Marco Kloos, Faisal H. M. Koua, Iñaki de Diego Martinez, Diogo Melo, Lukas Rane, Adam Round, Ekaterina Round, Abhisakh Sarma, Robin Schubert, Joachim Schulz, Marcin Sikorski, Mohammad Vakili, Joana Valerio, Jovana Vitas, Raphael de Wijn, Agnieszka Wrona, Ninon Zala, Arwen Pearson, Katerina Dörner, Giorgio Schirò, Elspeth F. Garman, András Lukács, Martin Weik

**Affiliations:** a Université Grenoble Alpes, CEA, CNRS, Institut de Biologie Structurale 38044Grenoble France; b European Molecular Biology Laboratory (EMBL) Hamburg Unit c/o DESY Notkestrasse 85 22607Hamburg Germany; c European XFEL Holzkoppel 4 22869Schenefeld Germany; d Department of Biophysics, Medical School University of Pecs Szigeti Street 12 7624Pécs Hungary; e Surrey Ion Beam Centre University of Surrey GuildfordGU2 7XH United Kingdom; f Institute for Nanostructure and Solid-State Physics Universität Hamburg HARBOR, Luruper Chaussee 149 22761Hamburg Germany; g Department of Biochemistry University of Oxford Dorothy Crowfoot Hodgkin Building, South Parks Road OxfordOX1 3QU United Kingdom; European Molecular Biology Laboratory, France

**Keywords:** bacterial adenylate cyclase, BLUF domain, AC domain, serial femtosecond crystallography, XFELs, synchrotrons, room-temperature X-ray crystallography, cryo-macromolecular crystallography, protein structure, light-activated enzymes, *Oscillatoria acuminata*

## Abstract

OaPAC is a recently discovered blue-light-using flavin adenosine dinucleotide (BLUF) photoactivated adenylate cyclase from the cyanobacterium *Oscillatoria acuminata* that uses adenosine triphosphate and translates the light signal into the production of cyclic adenosine monophosphate. Here, we report crystal structures of the enzyme in the absence of its natural substrate determined from room-temperature serial crystallography data collected at both an X-ray free-electron laser and a synchrotron, and we compare these structures with cryo-macromolecular crystallography structures obtained at a synchrotron by us and others. These results reveal slight differences in the structure of the enzyme due to data collection at different temperatures and X-ray sources. We further investigate the effect of the Y6W mutation in the BLUF domain, a mutation which results in a rearrangement of the hydrogen-bond network around the flavin and a notable rotation of the side chain of the critical Gln48 residue. These studies pave the way for picosecond–millisecond time-resolved serial crystallography experiments at X-ray free-electron lasers and synchrotrons in order to determine the early structural intermediates and correlate them with the well studied pico­second–millisecond spectroscopic intermediates.

## Abstract 2

Structures of the dark-adapted state of a photoactivated adenylate cyclase were determined from serial crystallography (SX) data collected at room temperature at an X-ray free-electron laser and a synchrotron, and are compared with cryo-macromolecular crystallography (MX) synchrotron structures obtained by us and others. These structures of the wild-type enzyme in combination with the cryo-MX synchrotron structure of a light-sensor domain mutant provide insight into the hydrogen-bond network rearrangement upon blue-light illumination and pave the way for the determination of structural intermediates of the enzyme by time-resolved SX.

## 1. Introduction

In the search for light-sensing proteins for optogenetic applications (Yawo *et al.*, 2021 ▸), photoactivated adenylate cyclases (PACs) have emerged as very promising candidates in the field, combining the function of a photoreceptor with that of an enzyme capable of second-messenger generation (Iseki & Park, 2021 ▸). First discovered as causing a change in the flagellar activity in the unicellular flagellate *Euglena gracilis* (Iseki *et al.*, 2002 ▸) via the activation of a protein kinase (Häder & Iseki, 2017 ▸) and later identified in both prokaryotes and eukaryotes, PACs are flavoproteins which accelerate the conversion of adenosine triphosphate (ATP) to cyclic adenosine monophosphate (cAMP) upon illumination with blue light (Iseki *et al.*, 2002 ▸; Stierl *et al.*, 2011 ▸; Penzkofer *et al.*, 2015 ▸; Raffelberg *et al.*, 2013 ▸; Blain-Hartung *et al.*, 2018 ▸; Ryu *et al.*, 2014 ▸, 2010 ▸). cAMP is a key second messenger in numerous signal transduction pathways, regulating various cellular functions including cell growth, differentiation and motility, gene transcription and protein expression (Gancedo, 2013 ▸). In cyanobacteria, cAMP that is produced after blue-light illumination has been shown to bind to a complex involved in the biogenesis of pili required for cell motility (Ohmori & Okamoto, 2004 ▸; Yoshimura *et al.*, 2002 ▸). Hence, cyanobacteria actively sense light gradients, which guide their locomotion, moving towards or away from the light direction (Hoiczyk, 2000 ▸). The potential to modulate the cellular concentration of cAMP is of great interest in the field of optogenetics (Ryu *et al.*, 2010 ▸; Fomicheva *et al.*, 2019 ▸; Stüven *et al.*, 2019 ▸).

OaPAC is a photoactivated adenylate cyclase from the cyanobacterium *Oscillatoria acuminata* (Ohki *et al.*, 2016 ▸) and it has a high sequence identity (57%) to the photoactivated adenylate cyclase bPAC from the soil bacterium *Beggiatoa* (Lindner *et al.*, 2017 ▸). OaPAC is a homodimer of a 366-residue protein comprising an N-terminal BLUF domain and a C-terminal class III adenylyl cyclase (AC) domain. BLUF domains contain a flavin adenine dinucleotide (FAD) chromophore and are ubiquitous blue-light sensors that are found in bacteria and unicellular eukaryotes. Upon blue-light illumination the hydrogen-bond network around the flavin is rearranged, resulting in a characteristic 10 nm red-shift in the main visible absorption band of the flavin (Park & Tame, 2017 ▸), with the signalling state decaying back in seconds to the dark-adapted state (Ohki *et al.*, 2017 ▸). This 10 nm red-shift is a feature that is not observed in other flavin-based photoreceptors (Chaves *et al.*, 2011 ▸; Losi *et al.*, 2015 ▸; Conrad *et al.*, 2014 ▸). The BLUF-regulated AC domain of OaPAC stimulates turnover of ATP up to 20-fold upon illumination (Ohki *et al.*, 2016 ▸; Blain-Hartung *et al.*, 2018 ▸). Cryo-macromolecular crystallography structures of OaPAC have been obtained in the dark-adapted state both in the presence and the absence of a nonhydrolyzable ATP analogue, as well as in the light-adapted state in the presence of the same analogue (Ohki *et al.*, 2016 ▸, 2017 ▸). Recently, room-temperature (RT) and cryo-structures of the dark-adapted state of OaPAC in complex with ATP have been solved by serial femtosecond crystallography (SFX; Boutet *et al.*, 2012 ▸) at an X-ray free-electron laser (XFEL; Chretien *et al.*, 2024 ▸) and by single-crystal cryo-macromolecular crystallography (cryo-MX) at a synchrotron (Chretien *et al.*, 2024 ▸). OaPAC has been shown to be catalytically active in the crystal form, as indicated by the characteristic 10 nm red-shift upon blue-light illumination and the increased cAMP levels measured in blue-light-exposed crystals soaked in buffer containing ATP and magnesium (Ohki *et al.*, 2017 ▸). Comparison of the light- and dark-adapted states has shown that the photoactivation mechanism *in crystallo* involves only very small movements, suggesting that very small but concerted shifts trigger the enzymatic activity tens of ångströms from the flavin chromophore (Ohki *et al.*, 2017 ▸). However, evidence for a major movement involved in the Trp90_in_/Met92_out_ switch has been shown in the recently published crystal structure of ATP-bound OaPAC solved from macrocrystals flash-cooled after 5 s of blue-light illumination at RT (Chretien *et al.*, 2024 ▸). These residues are the subject of an ongoing debate, with the tryptophan pointing out of the flavin-binding pocket and the methionine pointing inwards in some BLUF photoreceptors in the so-called Trp_out_ conformation, and vice versa in the Trp_in_ conformation, as summarized by Karadi *et al.* (2020 ▸). In addition, structures solved from microsecond time-resolved SFX (TR-SFX) data indicate significant changes around the flavin, with the rotation of the side chain of Gln48 (180° at 1.8 µs and 220° at 2.3 µs) being the most notable. This rotation is accompanied by a hydrogen-bond network rearrangement and destabilization of the loop region between the β5/β4 strands and the β5 strand/α3 helix (Chretien *et al.*, 2024 ▸). Changes in the vibrational modes of the protein backbone between the dark- and light-adapted states of OaPAC, in particular in the β5 strand of the BLUF domain, which is considered to be important for signal transduction, have previously been revealed by solution steady-state infrared difference spectroscopy (Tolentino Collado *et al.*, 2022 ▸). TR infrared measurements have pointed towards secondary-structural changes in the AC domain taking place beyond 100 µs (Tolentino Collado *et al.*, 2022 ▸). Indeed, a fast and a slow phase with time constants of 2.3 and 36 ms, respectively, have been revealed by transient absorption (TA) and transient grating (TG) experiments (Tokonami *et al.*, 2022 ▸; Nakasone *et al.*, 2023 ▸). These phases have been attributed to conformational changes in the BLUF domain and the α3 helix facilitated by Trp90, leading to an alteration in the relative orientation of the AC domains for functional activation.

Despite the present availability of several static structures of dark- and light-adapted states (Ohki *et al.*, 2016 ▸, 2017 ▸; Chretien *et al.*, 2024 ▸), structural intermediates on the microsecond timescale (Chretien *et al.*, 2024 ▸) and solution studies (Tolentino Collado *et al.*, 2022 ▸; Zhou *et al.*, 2022 ▸; Tokonami *et al.*, 2022 ▸; Nakasone *et al.*, 2023 ▸), the signal transduction pathway in OaPAC has not been revealed. TR-SFX is expected to play an essential role in filling this knowledge gap for two reasons. Firstly, it will allow access to the unexplored picosecond–nanosecond timescale (Brändén & Neutze, 2021 ▸; Weik & Domratcheva, 2022 ▸; de Wijn *et al.*, 2022 ▸; Khusainov *et al.*, 2024 ▸; Caramello & Royant, 2024 ▸) and, secondly, it will allow X-ray radiation damage-free data collection (Barends *et al.*, 2022 ▸; Garman & Weik, 2023 ▸). For both reasons, flavo­enzymes benefit from TR-SFX, as their structures are prone to radiation damage (Kort *et al.*, 2004 ▸), with the bending of the isoalloxazine moiety being used as an indicator of the reduction of the flavin (Røhr *et al.*, 2010 ▸). In addition, flavin radical intermediates are too short-lived to be identified at current synchrotron sources (Maestre-Reyna *et al.*, 2023 ▸; Christou *et al.*, 2023 ▸; Cellini *et al.*, 2024 ▸; Sorigué *et al.*, 2021 ▸).

Here, we report the first crystal structures of a substrate-free wild-type (WT) OaPAC obtained by RT serial crystallography at both an XFEL and a synchrotron. We compare these structures with substrate-free cryo-MX synchrotron structures of the WT and a mutant in which a tyrosine residue (Tyr6) which has a crucial role in the photoactivation process as the primary electron donor is replaced by a tryptophan residue. The latter structure shows the importance of this specific residue in the rearrangement of the hydrogen-bond network around the flavin. Comparison of our structures with those previously solved by other workers in the field also highlights the importance of the crystallization conditions, which may have an effect on secondary-structure elements in a protein. Since our crystallization conditions result in photoactive substrate-free OaPAC crystals, this paves the way for the characterization of light-induced structural intermediates.

## 2. Methods

### 2.1. Protein purification

Full-length wild-type (WT) OaPAC and Y6W mutant OaPAC were expressed and purified as described previously (Tolentino Collado *et al.*, 2022 ▸). WT OaPAC and Y6W OapAC plasmids were purchased from GenScript and both sequences were optimized using GenSmart. The WT OaPAC/pET-15b and Y6W OaPAC/pET-15b plasmids were transformed into *Escherichia coli* BL21(DE3) cells and grown on a Luria broth (LB) agar plate containing 100 µg ml^−1^ ampicillin. A single colony was used to inoculate 10 ml LB medium containing 100 µg ml^−1^ ampicillin and was grown at 37°C for 16 h or overnight. This culture was used to inoculate 1 l LB–ampicillin medium in a 4 l flask. The 1 l culture was incubated at 37°C until the optical density at 600 nm (OD_600_) reached 0.4–0.5, after which the temperature was decreased to 18°C followed by the addition of 1 m*M* isopropyl β-d-1-thiogalactopyranoside (IPTG) to induce protein expression. The cells were harvested after overnight incubation by centrifugation (6000*g*, 4°C) and the cell pellet was stored at −20°C. The cell pellet was then resuspended in lysis buffer containing protease-cocktail inhibitor, DNase (1 mg ml^−1^), 5 m*M* MgCl_2_ and lysozyme (1 mg ml^−1^). The resuspended cells were lysed by sonication and the cell debris was removed by centrifugation (39 000*g*, 30 min). The supernatant was loaded onto an Ni–NTA (Qiagen) column, which was washed with buffer containing 20 m*M* imidazole, and the protein was eluted with wash buffer containing 300 m*M* imidazole. The fractions containing protein were pooled together and purified to homogeneity using size-exclusion chromatography (Superdex 200), and the purity was assessed by SDS–PAGE. The protein concentration was determined using the extinction coefficients ɛ_280 nm_ = 29 870 *M*^−1^ cm^−1^ and ɛ_445 nm_ = 11 300 *M*^−1^ cm^−1^. The following buffers were used: lysis/wash buffer (50 m*M* Tris pH 8.0, 300 m*M* NaCl, 20 m*M* imidazole), elution buffer (50 m*M* Tris pH 8.0, 300 m*M* NaCl, 300 m*M* imidazole) and gel-filtration buffer (50 m*M* Tris pH 8.0, 150 m*M* NaCl).

### 2.2. Crystallization

Crystallization experiments were carried out at the High Throughput Crystallization Laboratory (HTX Lab) at the EMBL in Grenoble to find alternative crystallization conditions to those reported in the literature (Ohki *et al.*, 2016 ▸, 2017 ▸) in order to obtain OaPAC crystals (without substrate added) that diffracted to higher resolution (<2.9 Å resolution; Ohki *et al.*, 2016 ▸). OaPAC (7 mg ml^−1^) dissolved in 50 m*M* Tris pH 8.0, 150 m*M* NaCl crystallized in 0.06 *M* divalents (a mixture of magnesium chloride hexahydrate and calcium chloride dihydrate), 0.1 *M* Tris–Bicine pH 8.5, 30% (*v*/*v*) PEG 550 MME/PEG 20 000 at 20°C. Under these conditions, crystals reached dimensions of 90 × 50 × 20 µm^3^ within two days and were prepared for X-ray diffraction experiments using the CrystalDirect technology without cryoprotection (Zander *et al.*, 2016 ▸). Cryo-MX synchrotron data were collected at 100 K. Having identified initial macrocrystallization conditions, OaPAC microcrystals (without substrate added) were produced at 20°C using a batch crystallization procedure without seeding following a published approach (Shoeman *et al.*, 2023 ▸). 300 µl OaPAC (7 mg ml^−1^) was mixed with 700 µl crystallization solution [0.06 *M* divalents, 0.1 *M* Tris–Bicine pH 8.5, 30% (*v*/*v*) PEG 500 MME/PEG 20 000] and left in the dark. The size of the microcrystals (<15 × 15 × 5 µm) was estimated using a Discovery V12 Stereo Zeiss microscope.

### 2.3. Data collection and processing, structure solution and refinement

**Figure 1:**
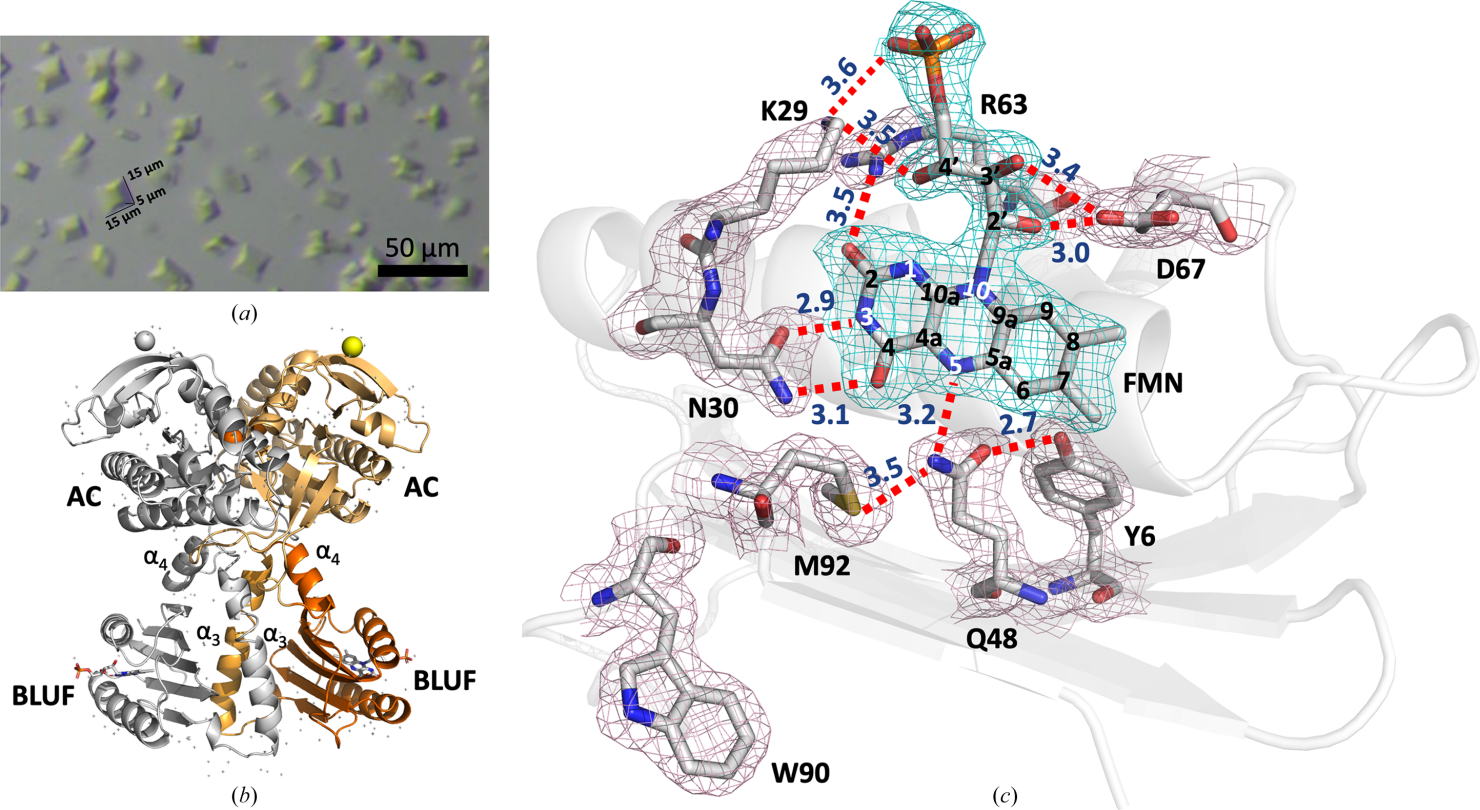
(*a*) WT OaPAC crystals used at the EuXFEL. Crystal size <15 × 15 × 5 µm^3^; the scale bar corresponds to 50 µm. (*b*) Ribbon diagram of the SFX structure of WT OaPAC; monomeric OaPAC, grey; BLUF domain, orange; α_3_ helix, light orange; α_4_ helix, bright orange; AC domain, yellowish-orange; calcium ions are represented by spheres. (*c*) Flavin active site of OaPAC in the SFX structure (PDB entry 9f1w) showing key amino-acid residues participating in the hydrogen-bond network. 2*mF*_o_ − *DF*_c_ electron-density maps are represented as blue (flavin) and light purple (residues) meshes at a contour level of 1.0σ.

SFX experiments were carried out with the SPB/SFX instrument (Mancuso *et al.*, 2019 ▸) at the European XFEL, Schenefeld, Germany (proposal PCS 3018, 28–29 October 2022, 4.5 h). The OaPAC microcrystal jetting conditions were tested in the injection laboratory at the IBS in Grenoble and in the XFEL Biology Infrastructure (XBI) laboratory (Han *et al.*, 2021 ▸) before the beamtime. A total volume of 4.5 ml OaPAC microcrystal slurry [10%(*v*/*v*) gravity-settled crystals; Fig. 1 ▸(*a*)] was injected into the Interaction Region Upstream (IRU) under high vacuum with a standard gas dynamic virtual nozzle (GDVN; 75 µm inner diameter; Vakili *et al.*, 2022 ▸). The GDVN was equipped with a mesh (30 × 34 µm) inline sample filter and operated at a liquid flow rate of 30 µl min^−1^ (corresponding to a jet velocity of 45 m s^−1^). The sample was probed using trains delivered at 10 Hz with a 564 kHz intra-train repetition rate [9.3 keV photon energy, 50 fs (FWHM) pulse length, ∼2.5 mJ pulse energy], each containing 180 pulses spaced by 1.773 µs in time. The X-ray beam focal size was 3.5 µm in diameter (FWHM) and the time interval between each 180-pulse train was ∼100 ms. Radiation damage-free diffraction data (Nass *et al.*, 2021 ▸) from OaPAC crystals were recorded using a 1 megapixel Adaptive Gain Integrating Pixel Detector (AGIPD; Allahgholi *et al.*, 2019 ▸). To avoid sample damage by the preceding pulses (Stan *et al.*, 2016 ▸; Grünbein *et al.*, 2021 ▸), an optical imaging system with a femtosecond optical laser for jet illumination was used to observe the effects of the interaction with the XFEL pulses and to make sure that the liquid jet had time to recover between two successive X-ray pulses.

Hits were identified using *NanoPeakCell* (Coquelle *et al.*, 2015 ▸) and were saved into HDF5 files for further processing (indexing and integration) with *CrystFEL* version 0.10.1 (White, 2019 ▸). A total of 17 434 418 raw images were collected with a hit rate of 1.6% (number of hits 285 671), consuming 4.5 ml of the microcrystal suspension. Diffraction spots were detected at a resolution better than 1.75 Å. 27 202 lattices were indexed (indexing rate of 9.5%) in space group *P*2_1_2_1_2, with unit-cell parameters *a* = 102.9, *b* = 54.9, *c* = 73.0 Å, α = β = γ = 90° (Table 1 ▸). By inspection of the *R*_split_ and CC_1/2_ values, the high-resolution limit was determined to be 1.75 Å.

For structure refinement, starting phases were obtained by molecular replacement using *Phenix* (Liebschner *et al.*, 2019 ▸). The search model was PDB entry 4yut (Ohki *et al.*, 2016 ▸) after removing the flavin mononucleotide (FMN; an isoalloxazine ring with a ribityl and one phosphate group). It should be noted that although BLUF domains are assumed to contain FAD *in vivo* (Laan *et al.*, 2004 ▸), electron density corresponding to the adenine moiety of FAD has not been identified in OaPAC expressed in *E. coli*; FMN is instead found in the electron density (Ohki *et al.*, 2016 ▸), similar to observations for other BLUF domains (AppA and bPAC; Anderson *et al.*, 2005 ▸; Jung *et al.*, 2006 ▸; Lindner *et al.*, 2017 ▸). Refinement in real and reciprocal space, electron-density map calculations and solvent-molecule assignments were performed using *Phenix*. The occupancies of the alternate conformations found in several residues were refined using occupancy refinement in *Phenix* after the initial values were set to equal occupancy (0.5) for each conformer. Model building and real-space refinement were performed using *Coot* (Emsley *et al.*, 2010 ▸). During the refinement process, water molecules, FMN and calcium ions were added stepwise to the model. *PyMOL* (Schrödinger) was used to prepare figures. Data-collection and refinement statistics are summarized in Table 1 ▸. Atomic coordinates and structure-factor amplitudes have been deposited in the PDB (accession code 9f1w; Table 1 ▸).

The serial synchrotron crystallography (SSX) experiment was conducted at RT on EMBL beamline P14-2 (T-REXX) at the PETRA III synchrotron, DESY, Hamburg using a 12.7 keV beam with a flux of 1.2 × 10^12^ photons s^−1^ and a beam size of 10 × 7 µm^2^ (horizontal × vertical, FWHM). The 15 × 15 × 5 µm^3^ microcrystals were loaded onto HARE chips and mounted on the translation-stage holders as described previously (Mehrabi *et al.*, 2020 ▸). Briefly, ∼100 µl of a slurry of crystals (1.0–1.5 × 10^6^ crystals per millilitre; assessed using a Neubauer cell-counting chamber) was placed onto the chips using a pipette, and a laboratory vacuum pump equipped with a dial gauge was used to suck the crystals into the wells of the chips via a chip-loading block. The chip-loading procedure took place inside a humidity hood with an automatic humidity control and under red-light conditions. Diffraction images were processed automatically at the beamline using *CrystFEL* version 0.10.2 (White, 2019 ▸) with the *XGANDALF* indexing routine (Gevorkov *et al.*, 2019 ▸).

Diffraction data from the WT and Y6W OaPAC single crystals (90 × 50 × 20 µm^3^) were collected at 100 K on the ID30A-1 beamline at the ESRF, Grenoble (IBS-BAG mx2398). The SSX structure of WT OaPAC was solved by molecular replacement using *DIMPLE* (from the *CCP*4 suite; Agirre *et al.*, 2023 ▸), whereas the search models and refinement protocols for the SSX (PDB entries 9f1x) and cryo-MX models (PDB entries 9f1y and 9f1z) were similar to those used for the SFX structure, as described above.

### 2.4. Micro-PIXE

Proton-induced X-ray emission (PIXE) analysis of proteins was carried out at the Ion Beam Centre, University of Surrey, United Kingdom using the methods detailed in Grime & Garman (2023 ▸). In summary, characteristic X-ray emission was induced using a 2.5 MeV proton beam of less than 2 µm in diameter incident under vacuum on dried OaPAC crystals that had been washed in a metal-free buffer (volume per droplet ∼0.1 µl) before mounting.

The emitted X-rays were detected using a silicon drift detector (Rayspec Ltd, United Kingdom). In order to detect sodium and magnesium, we removed the 130 µm thick beryllium filter that is normally used to avoid the deleterious effects of recoiling protons. Additionally, backscattered protons were detected using a silicon surface barrier particle detector and the resulting spectrum was analysed to obtain the sample thickness and gross matrix composition. By scanning the proton beam in *x* and *y* over the dried sample, spatial maps were obtained of all elements with *Z* ≥ 11 (sodium) present in the sample. Quantitative information was obtained by collecting three- or four-point spectra from each droplet. PIXE spectra were processed with *GUPIX* (Campbell *et al.*, 2021 ▸) using the matrix composition derived from the simultaneously collected Rutherford backscattered proton spectrum. The concentrations of the elements were used to calculate the relative amount of each element per protein molecule in the sample using the sulfur signal from the known number of cysteines and methionines in the protein sequence as an internal standard for normalization.

### 2.5. UV–visible absorption spectroscopy on OaPAC macrocrystals

To examine the photoactivity of OaPAC crystals in the specific crystallization conditions used here, we recorded the absorption spectra of OaPAC crystals in the dark- and light-adapted states [Supplementary Fig. S1(*a*)] using the Cal(ai)^2^doscope, a custom-made microspectrophotometer (Byrdin & Bourgeois, 2016 ▸; Rane *et al.*, 2023 ▸). An OaPAC crystal (90 × 50 × 20 µm) was mounted between two microscope slides and absorption spectra were recorded at RT in the dark-adapted state and in the light-adapted state after illumination with a continuous-wave 488 nm laser [illumination for 5 s, 1 mW power, top hat 100 µm (FWHM) laser spot]. Absorption spectra of the dark- and light-adapted states were also recorded for OaPAC in solution with a SPECORD S600 (AnalytikJena) spectrophotometer [Supplementary Fig. S1(*b*)].

## 3. Results and discussion

### 3.1. Room-temperature SFX structure of substrate-free OaPAC

Table 2 ▸ summarizes all the available structures of OaPAC in the dark-adapted state. For this work, the SFX structure of substrate-free OaPAC (PDB entry 9f1w) was solved at 1.75 Å resolution [Fig. 1 ▸(*b*)]. OaPAC crystals [Fig. 1 ▸(*a*)] were grown in the dark in a crystal form (*P*2_1_2_1_2; unit-cell parameters *a* = 102.9, *b* = 54.9, *c* = 73.0 Å, α = β = γ = 90°) with one monomer in the asymmetric unit. Fig. 1 ▸(*b*) shows a ribbon model of the characteristic parallel dumbbell-shaped dimer with an antiparallel arrangement of the N-terminal BLUF and C-terminal AC domains, as described previously for cryo-MX synchrotron structures (Ohki *et al.*, 2016 ▸, 2017 ▸; Chretien *et al.*, 2024 ▸; PDB entries 4yut, 4yus and 8qfe) and for the SFX structure of ATP-bound OaPAC (Chretien *et al.*, 2024 ▸; PDB entry 8qfh). The BLUF domains dimerize through their α3_BLUF_-capping helices that form an intermolecular coiled coil, whereas a four-helix bundle consisting of the C-termini of the α3 capping helices, the α4_BLUF_ helix and the α4_BLUF_–β1_AC_ loop is formed on which the AC domains reside. The central coiled-coil domain has been suggested to play a key role in the transduction of the signal received by the BLUF domain (Ohki *et al.*, 2016 ▸).

**Figure 2:**
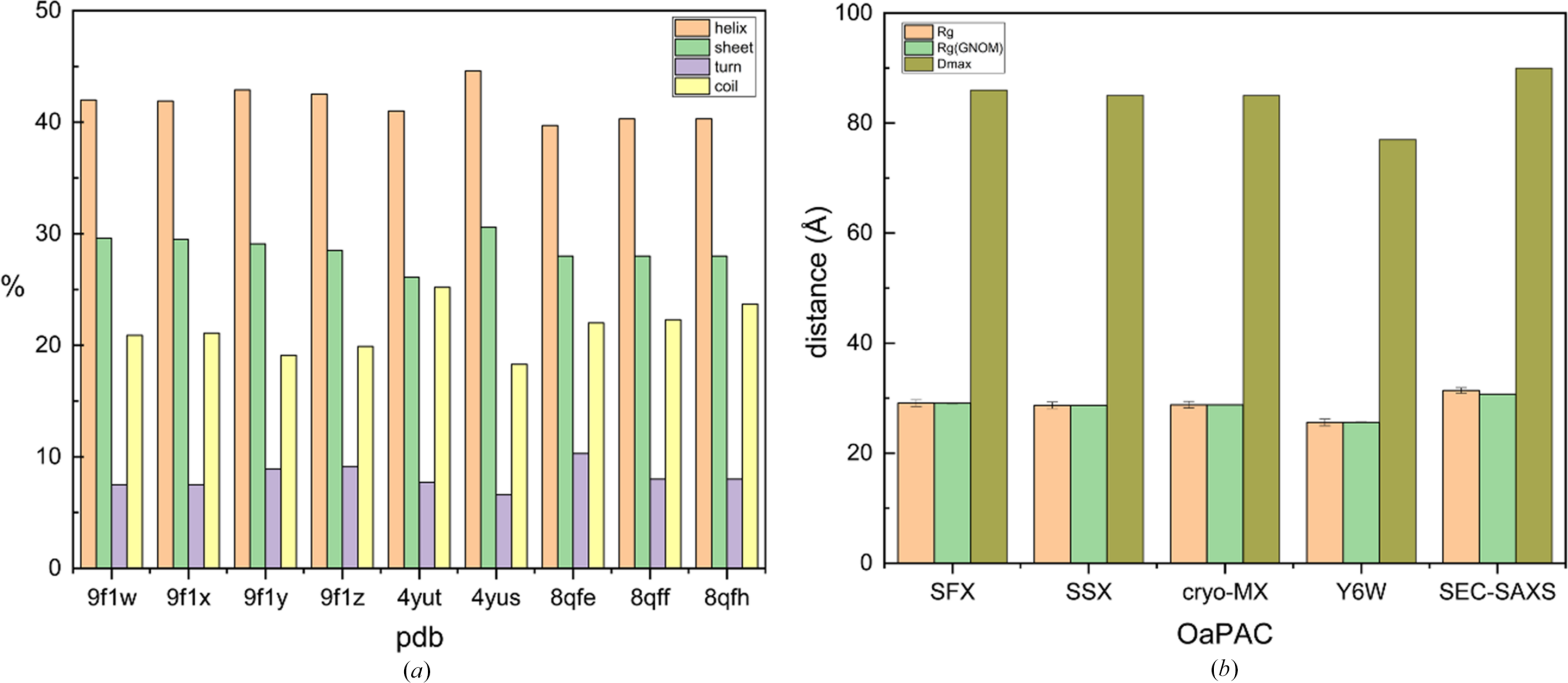
(*a*) Assignment of secondary-structure elements in all OaPAC structures. (*b*) The basic SAXS parameters *R*_g_ (radius of gyration; orange), *D*_max_ (maximum size; olive) and *R*_g_(*GNOM*) (*R*_g_ calculated using *GNOM*; Svergun, 1992 ▸; green) derived from the theoretical SAXS profiles of the OaPAC structures (Supplementary Fig. S3) calculated for all four OaPAC structures are presented and shown as bars. SEC-SAXS data are from Ujfalusi-Pozsonyi *et al.* (2024 ▸). *GNOM* (Svergun, 1992 ▸) is an indirect transform program for small-angle scattering data processing. It reads in one-dimensional scattering curves (possibly smeared with instrumental distortions) and evaluates the particle distance distribution function *P*(*r*). For *R*_g_, *R*^2^ is 1.0 for all structures and 0.9908 for SEC-SAXS, and for *D*_max_ χ^2^ = 1.88 × 10^−7^ for WT SFX, 2.31 × 10^−7^ for WT SSX, 2.04 × 10^−7^ for WT cryo-MX, 1.27 × 10^−7^ for Y6W cryo-MX and 1.0728 for SEC-SAXS.

In earlier studies, OaPAC was crystallized in orthorhombic (PDB entry 4yut) and hexagonal (PDB entry 4yus) crystal forms (Ohki *et al.*, 2016 ▸, 2017 ▸). In the latter, the best resolution was achieved in the presence of a non­hydrolyzable ATP analogue [ApCpp, adenosine-5′-[(α,β)-methyleno]triphos­phate], although no electron density was observed for this analogue. Yet another space group has recently been reported for the cryo-MX synchrotron structure of OaPAC in the absence of substrate (PDB entry 8qfe), for the cryo-MX structure of ATP-bound OaPAC (PDB entry 8qff) and the SFX structure of ATP-bound OaPAC (PDB entry 8qfh). We attribute the differences in the space groups and unit cells to the different crystallization conditions used. In addition, subtle differences are observed in the percentages of the secondary-structure elements in our WT OaPAC SSX (PDB entry 9f1x) and WT OaPAC cryo-MX (PDB entries 9f1y, 4yut and 8qfe) structures and our SFX substrate-free OaPAC model (PDB entry 9f1w). Using *YASARA* (https://www.yasara.org), we calculated the following contributions for PDB entry 9f1w: 42% helix, 29.6% sheet, 7.5% turn, 20.9% coil. These values are slightly, but not significantly, different from those calculated for the cryo-structures (PDB entry 9f1y: 42.9% helix, 29.1% sheet, 8.9% turn, 19.1% coil; PDB entry 4yut: 41% helix, 26.1% sheet, 7.7% turn, 25.2% coil; PDB entry 4yus: 44.6% helix, 30.6% sheet, 6.6% turn, 18.3% coil; PDB entry 8qfe: 39.7% helix, 28% sheet, 10.3% turn, 22% coil) and are all summarized in Fig. 2 ▸(*a*) and Supplementary Table S1.

Upon blue-light illumination, crystals of OaPAC have previously been shown to produce a characteristic red-shift and to turn over ATP (Ohki *et al.*, 2017 ▸). Using our custom-made microspectrophotometer [Cal(ai)^2^doscope; Byrdin & Bourgeois, 2016 ▸; Rane *et al.*, 2023 ▸], we examined the photoactivity of the OaPAC crystals produced by our specific crystallization conditions. Supplementary Fig. S1(*a*) shows the absorption spectra of an OaPAC crystal in the dark- and light-adapted states. The light-adapted state is characterized by the typical red-shift observed for OaPAC in solution [Supplementary Fig. S1(*b*)]. The absorption spectrum of the oxidized flavin of OaPAC in solution exhibits two broad absorption bands, with peaks at 375 nm for *S*_0_→*S*_2_ and 444 nm for *S*_0_→*S*_1_ (Stanley & MacFarlane, 2000 ▸), which red-shifts upon blue-light excitation as observed in BLUF domains. The similar red-shift observed in our OaPAC crystals [Supplementary Fig. S1(*a*)] suggests that the specific crystallization condition produces crystals that are photoactive and hence suitable for time-resolved crystallographic experiments. Such studies will allow a correlation of the available spectroscopic signatures for the substrate-free enzyme (Tolentino Collado *et al.*, 2022 ▸, 2024 ▸) with the early-formed structural intermediates. It should be pointed out that this is the first time that photoactivity has been reported for substrate-free OaPAC crystals. Previous preparations of photoactive OaPAC crystals contained ApCpp (Ohki *et al.*, 2017 ▸) and ATP (Chretien *et al.*, 2024 ▸), with structural intermediates from ATP-bound OaPAC crystals reported on the microsecond timescale (Chretien *et al.*, 2024 ▸).

### 3.2. Structural description of the BLUF domain of the SFX structure of substrate-free OaPAC

The overall fold of the BLUF domain in the RT SFX crystal structure of substrate-free OaPAC (PDB entry 9f1w) reveals the characteristic compact arrangement of BLUF domains. It consists of a five-stranded mixed β-sheet with two α-helices running parallel to the β-strands on one side and the helix–turn–helix unit on the other side of the sheet. The first 96 amino-acid residues of the protein form an αβ sandwich with a typical ferredoxin-like β_1_α_1_β_2_β_3_α_2_β_4_ topology [Fig. 1 ▸(*b*)]. Fig. 1 ▸(*c*) shows key, highly conserved residues around the flavin which form an intricate hydrogen-bond network that is of functional significance. Upon blue-light illumination, a characteristic rearrangement occurs (as indicated by the absorption and vibrational spectra of the flavin) which is known to trigger signal transduction from the BLUF domain to the effector domain of the protein (Lukacs *et al.*, 2022 ▸). We observe clear electron density for the flavin and the ribityl side chain, but no apparent density for the adenine moiety, similar to previous observations in OaPAC (Ohki *et al.*, 2016 ▸) and other BLUF domains (AppA and bPAC; Anderson *et al.*, 2005 ▸; Jung *et al.*, 2006 ▸; Lindner *et al.*, 2017 ▸). In the flavin binding environment we identify the conserved residues Tyr6 and Gln48 that play a key role in the photoactivation mechanism (Tolentino Collado *et al.*, 2022 ▸). The amino group of Gln48 forms hydrogen bonds to Met92 (3.5 Å) and N5 of the flavin (3.2 Å), whereas the carbonyl group forms a hydrogen bond to the hydroxyl group of Tyr6 (2.7 Å). Other residues that are part of the extensive hydrogen-bond network in the active site are (i) Asn30, which hydrogen-bonds to the C4=O carbonyl (3.1 Å) and N3 of the FMN (2.9 Å), (ii) Arg63, which hydrogen-bonds via its amino group to the C2=O carbonyl of the FMN (3.5 Å), (iii) Lys29, which hydrogen-bonds to one O atom of the phosphate group of the FMN (3.6 Å) and the O atom on C4′ of the ribityl chain of the FMN (3.5 Å), and (iv) Asp67, which hydrogen-bonds to the O atoms on C2′ (3.0 Å) and C3′ (3.4 Å) of the ribityl chain of the FMN.

### 3.3. Structural description of the AC domain of the SFX structure of substrate-free OaPAC

**Figure 3:**
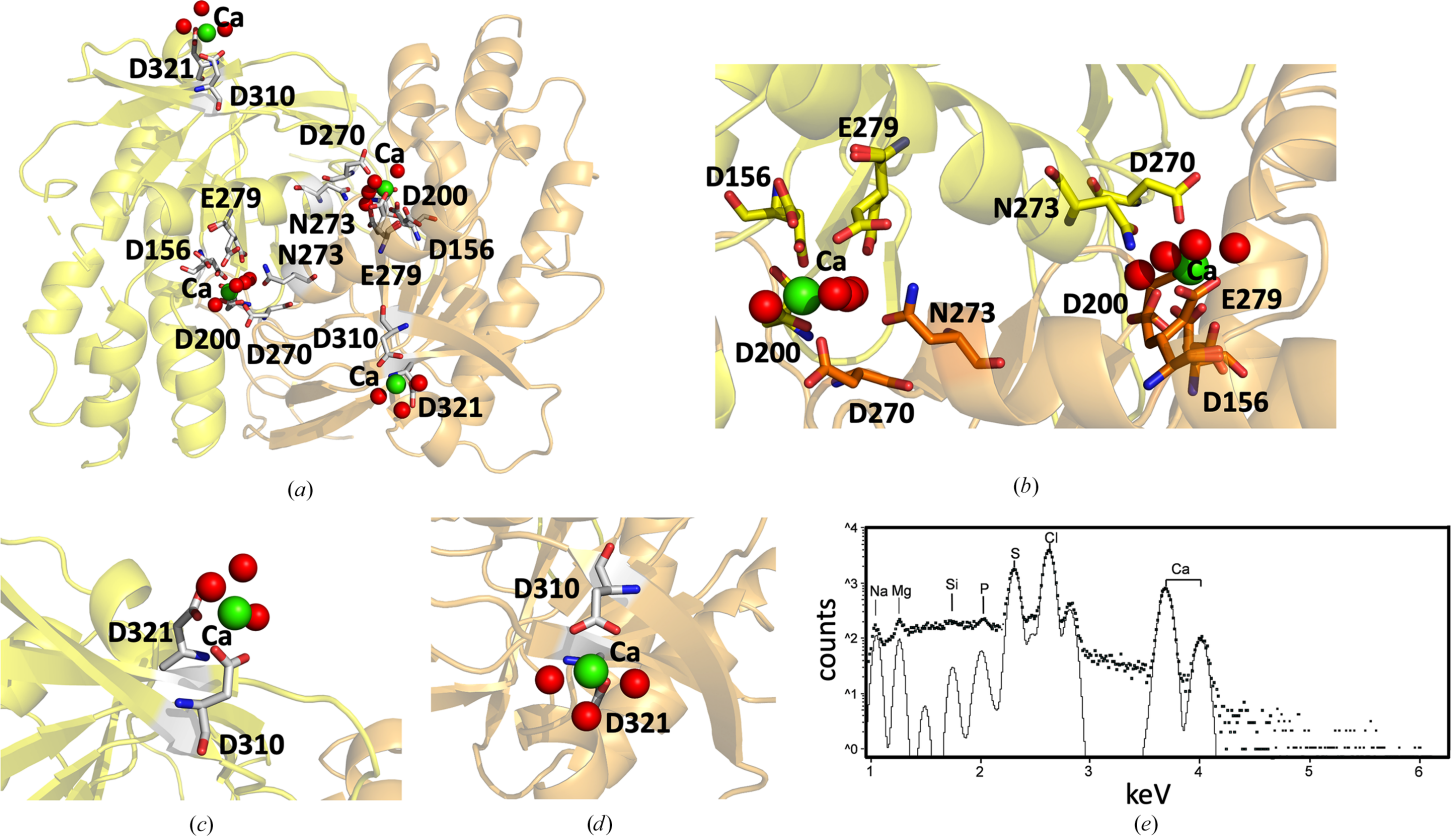
(*a*) AC domains in WT OaPAC (SFX; PDB entry 9f1w) showing the metal ion binding sites and surrounding residues; green spheres represent calcium ions and red spheres represent water molecules. (*b*) Zoom in on the dimer interface and the internal calcium binding sites with coordinating residues. (*c*, *d*) Zoom in on the calcium binding sites on the surface of WT OaPAC. (*e*) Micro-PIXE analysis of WT OaPAC crystals confirms the presence of calcium and magnesium ions in the crystals.

The AC domains in OaPAC exhibit the canonical type III fold and form a homodimer with two active sites at the dimer interface [Fig. 3 ▸(*a*)]. In class III AC domains the catalytic centres are formed at the dimer interface, employing a two-metal-ion mechanism where one metal ion coordinates to the triphosphate moiety of ATP while the other ion activates the 3-hydroxy group for nucleophilic attack on the α-phosphate (Linder, 2006 ▸). Fig. 3 ▸(*b*) shows two areas at the dimer interface where electron density (not shown) exists between Asp156/Asp200/Glu279 (chain *A*) and Asp270/Asn273 (chain *B*) and between Asp156/Asp200/Glu279 (chain *B*) and Asp270/Asn273 (chain *A*). Based on the ATP binding site arrangement in Y7F bPAC (Lindner *et al.*, 2017 ▸) and in class III AC domains (Steegborn, 2014 ▸), this electron density (not shown) can be attributed to the two metal ion binding sites. Indeed, the recently published ATP-bound structure of OaPAC (PDB entry 8qff) confirms the involvement of Asp156, Asp200 and Glu279 in the ATP binding site, which requires two magnesium ions when ATP is bound and one in the ATP-free form (Chretien *et al.*, 2024 ▸). Besides these two metal ion binding sites in our SFX structure of the substrate-free OaPAC (PDB entry 9f1w), we also observe electron density (not shown) around Asp321/Asp310 for both chains *A* and *B* at the surface of the AC domains [Figs. 3 ▸(*c*) and 3 ▸(*d*)].

Although our crystallization conditions contain both magnesium and calcium, our experimental PIXE results and theoretical calculations (*CheckMyMetal*; Gucwa *et al.*, 2023 ▸), as discussed below, suggest that only calcium is present in the binding sites of the AC domains. In particular, Fig. 3 ▸(*e*) shows the micro-PIXE analysis of WT OaPAC crystals, which have an average of two Mg atoms and 11 Ca atoms per protein molecule. The signal for the point spectra for magnesium was between three and six times above the limit of detection (LOD) and that for calcium was between 50 and 100 times above the LOD. The *y* axis is displayed on a log_10_ scale to help to visualize trace elements. Characteristic peaks for S, Cl and Ca atoms are highlighted. The black line shows the fit to these peaks following background subtraction. In addition, the *CheckMyMetal* validation server (Gucwa *et al.*, 2023 ▸) favours the presence of a Ca atom in each one of the four binding sites in the biological dimer, whereas modelling of magnesium results in large positive *mF*_o_ − *DF*_c_ peaks (Supplementary Fig. S2), suggesting that ions with more electrons are actually present. Based on the above analysis, we propose that the Mg ions identified in the micro-PIXE analysis may be present in channels of the crystal. Although Mg ions have been observed in the ATP binding site of the recently reported SFX structure of ATP-bound OaPAC (PDB entry 8qfh; Chretien *et al.*, 2024 ▸), it should be pointed out that no Ca ions were present in their crystallization conditions. Calcium is a nontransition element which demonstrates a variety of coordination numbers, with six to eight being the most usual values, and as a ‘hard’ metal ion (Ca^2+^) prefers ‘hard’ ligands with low polarizability, with oxygen being the most preferable coordinating atom (Dudev & Lim, 2003 ▸). The identified Ca^2+^ ions coordinate with four water molecules and five residues (Asp156, Asp200, Asp270, Asn273 and Glu279) in the internal metal binding site, whereas on the surface they coordinate with three water molecules and two residues (Asp310 and Asp321) [Figs. 3 ▸(*b*), 3 ▸(*c*) and 3 ▸(*d*)]. It should be mentioned that the calcium ion here is not physiologically relevant and although magnesium is also found in our structure, it is not the metal ion that is found in the vicinity of the ATP binding sites. Furthermore, the calcium ion does not have a structural role either, as OaPAC has been reported to retain its dimeric form in the absence of calcium (Ohki *et al.*, 2016 ▸; Ujfalusi-Pozsonyi *et al.*, 2024 ▸).

### 3.4. Comparison of the SFX structure of substrate-free OaPAC with the SSX and cryo-synchrotron structures of substrate-free OaPAC

**Figure 4:**
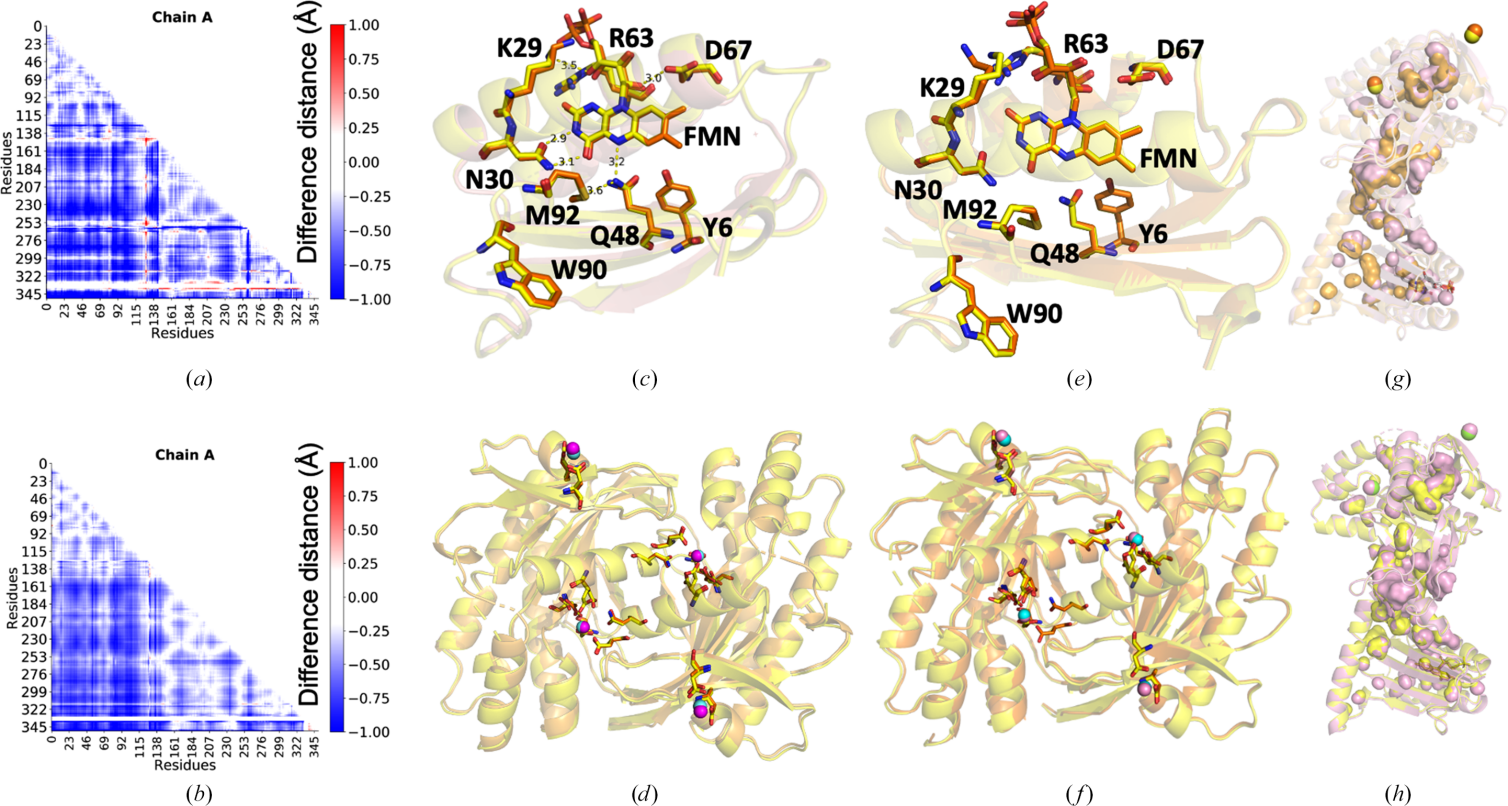
(*a*) Distance difference matrix (DDM) calculated between the WT OaPAC SFX structure (PDB entry 9f1w) and the WT OaPAC cryo-MX synchrotron structure (PDB entry 9f1y). Red and blue indicate increasing and decreasing distances with respect to the WT OaPAC SFX structure, respectively. (*b*) Distance difference matrix calculated between the SFX structure and the SSX structure of WT OaPAC. Red and blue indicate increasing and decreasing distances compared with the SFX structure, respectively. Residues 332–337 are not resolved in the SFX structure, which results in the white area in this region in the DDM (see Supplementary Note S1). (*c*) Superimposition of the BLUF domains of the WT OaPAC SFX structure (yellow) and the WT OaPAC cryo-MX synchrotron structure (orange). Distances (in Å) are shown for the WT OaPAC SFX structure. (*d*) Superimposition of the AC domains of the WT OaPAC SFX structure (yellow) and the WT OaPAC cryo-MX synchrotron structure (orange) showing amino-acid residues coordinated to the calcium ions (SFX, pink spheres; cryo, cyan spheres). (*e*) Superimposition of the BLUF domains of the WT OaPAC SFX structure (yellow) and of the WT OaPAC SSX structure (PDB entry 9f1x; orange). (*f*) Superimposition of the AC domains of the WT OaPAC SFX structure (yellow) and of the WT OaPAC SSX structure (orange) showing amino-acid residues coordinated to the calcium ions (SFX; pink spheres, SSX; cyan spheres). (*g*) Superimposition of WT OaPAC SFX and cryo-MX synchrotron monomer structures (SFX, pink; cryo-MX, orange; calcium ions in SFX, orange spheres; calcium ions in cryo-MX, yellow spheres) showing cavities calculated with *PyMOL*. (*h*) Superimposition of WT OaPAC SFX and SSX monomer structures (SFX, pink; SSX, yellow; calcium ions in SFX, pink spheres; calcium ions in SSX, green spheres) showing cavities calculated with *PyMOL*.

Our WT OaPAC SFX structure (PDB entry 9f1w) displays a fold similar to the WT OaPAC SSX structure (0.364 Å C_α_ r.m.s.d. over 350 residues; PDB entry 9f1x) and to the WT OaPAC cryo-MX synchrotron structure (0.446 Å C_α_ r.m.s.d. over 350 residues; PDB entry 9f1y). They also share the same space group (*P*2_1_2_1_2; Table 1 ▸) and there are no significant changes in the contribution of the secondary-structure elements [Fig. 2 ▸(*a*), Supplementary Table S1]. Similarly, calculation of the theoretical SAXS parameters derived from the crystal structures using the *FoXS* server (Schneidman-Duhovny *et al.*, 2016 ▸) does not reveal significant differences in the radius of gyration *R*_g_ and the maximum particle dimension *D*_max_, which are in line with the recently reported values in solution (Ujfalusi-Pozsonyi *et al.*, 2024 ▸) [Fig. 2 ▸(*b*), Supplementary Fig. S3]. In order to reveal any potential differences between these structures, we have calculated the distance difference matrices (DDMs), which highlight changes in interatomic distances. The red and blue colours in Figs. 4 ▸(*a*) and 4 ▸(*b*) indicate an increase or decrease in distances, respectively, compared with the reference structure (Seno & Gō, 1990 ▸). The DDMs reveal that the WT OaPAC cryo-MX synchrotron (PDB entry 9f1y) and WT OaPAC SSX (PDB entry 9f1x) structures are more compact than the WT OaPAC SFX (PDB entry 9f1w) structure [Figs. 4 ▸(*a*) and 4 ▸(*b*)]. Comparisons of the BLUF and AC domains of the WT OaPAC SFX structure (PDB entry 9f1w) with those of the WT OaPAC cryo-MX synchrotron (PDB entry 9f1y) and WT OaPAC SSX (PDB entry 9f1x) structures are shown in Figs. 4 ▸(*c*) and 4 ▸(*d*) and in Figs. 4 ▸(*e*) and 4 ▸(*f*), respectively. The only notable differences in the SFX structure compared with the cryo and SSX structures are that the side chain of Lys29 in the BLUF domain is in a different orientation and, in the AC domain, Asp156 and Glu279 (Fig. 4 ▸) display two conformations at RT. In addition, Supplementary Fig. S4 shows an enlarged view of the coordination of the internal calcium ions with residues which display a double conformation in the SFX structure of OaPAC. The WT OaPAC structure (PDB entry 9f1w) is almost identical to the WT OaPAC SSX structure (PDB entry 9f1x) as hardly any differences are observed [Figs. 4 ▸(*e*) and 4 ▸(*f*)]. Small differences are seen in the internal cavities of the WT OaPAC SFX structure (PDB entry 9f1w) compared with the WT OaPAC SSX (PDB entry 9f1x) and the WT OaPAC cryo-MX (PDB entry 9f1y) structures, as illustrated in Figs. 4 ▸(*g*) and 4 ▸(*h*). It should also be noted that the number of water molecules in the WT OaPAC cryo-MX synchrotron structure is higher compared with that in the RT structures (WT OaPAC SFX and WT OaPAC SSX structures; Table 1 ▸). This is to be expected as at low temperature the water molecules become more observable in the electron-density maps because of the decreased average flexibility of all atoms (Carugo, 2016 ▸) and the lower available thermal dynamic energy. It should also be mentioned that the present work is not a systematic study of the RT structures of OaPAC, and as a result a statistical analysis for a quantitative comparison is not presented here. However, the lack of significant differences between the WT OaPAC SFX and WT OaPAC SSX data are in line with a systematic study on other proteins by Mehrabi *et al.* (2021 ▸) which has shown that SFX and SSX measurements can result in data of equivalent quality.

### 3.5. Comparison of the WT and Y6W mutant OaPAC structures obtained by cryo-MX

**Figure 5:**
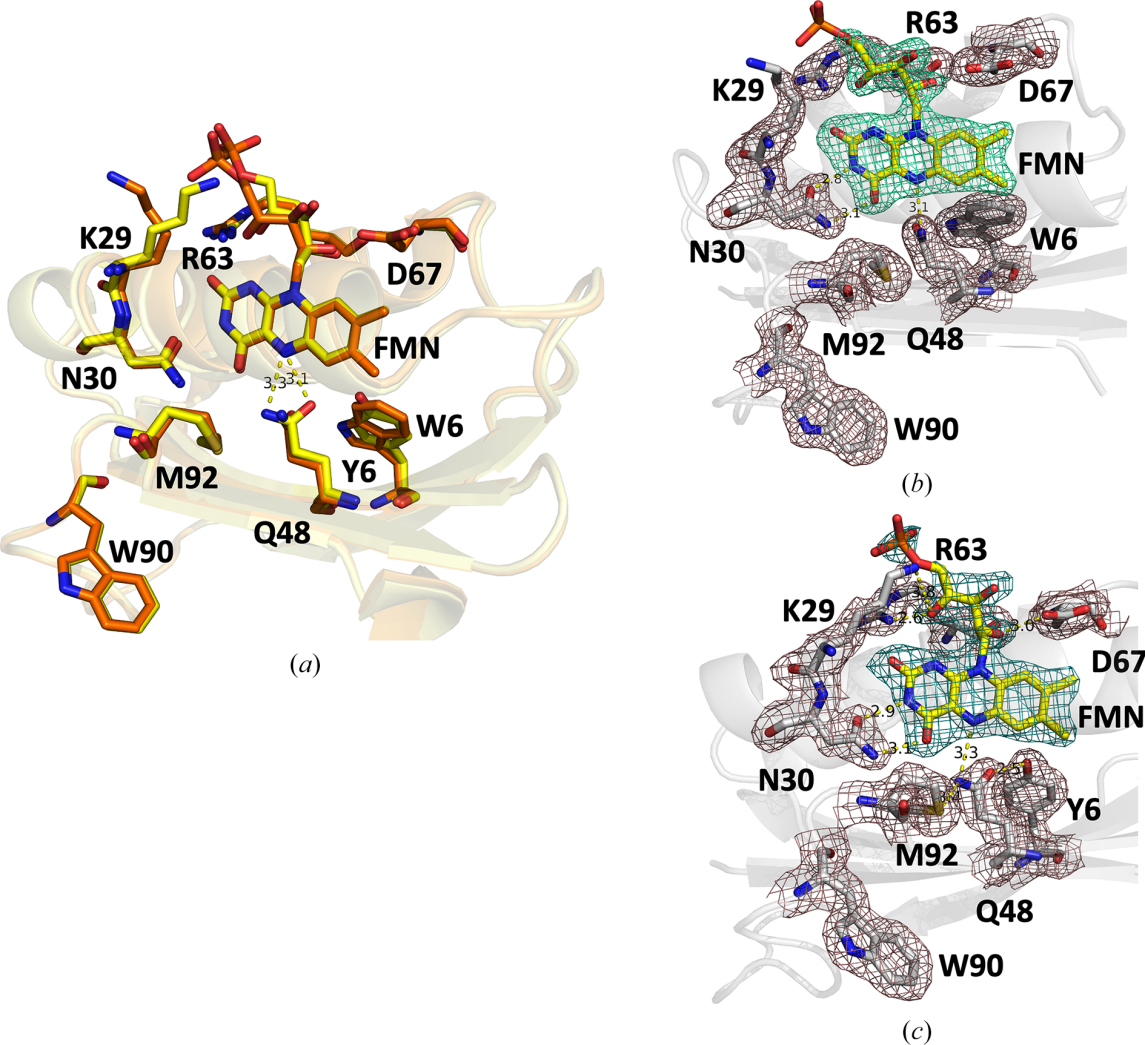
(*a*) Superimposition of the BLUF domain of the cryo-MX synchrotron structures of WT OaPAC (yellow; PDB entry 9f1y) and Y6W OaPAC (orange; PDB entry 9f1z). Distances (in Å) are shown for the cryo-MX synchrotron structure of OaPAC. (*b*) Flavin active site of Y6W OaPAC in the Y6W OaPAC cryo-MX synchrotron structure showing amino-acid residues participating in the hydrogen-bond network. 2*mF*_o_ − *DF*_c_ maps are represented as blue (flavin) and purple (residues) meshes at a contour level of 1.0σ. (*c*) Flavin active site of WT OaPAC in the WT OaPAC cryo-MX synchrotron structure showing amino-acid residues participating in the hydrogen-bond network. 2*mF*_o_ − *DF*_c_ maps are represented as blue (flavin) and purple (residues) meshes at a contour level of 1.0σ.

Tyr6 plays a key role in the photoactivation mechanism of OaPAC (Tolentino Collado *et al.*, 2022 ▸) and of BLUF domains in general (Park & Tame, 2017 ▸). In OaPAC, a concerted proton-coupled electron transfer (PCET) from Tyr6 which forms the neutral flavin radical takes place, followed by a recombination of the radical species that results in the hydrogen-bond rearrangement around the flavin and transduction of the light signal to the AC domains. Mutation of the analogous tyrosine residue in AppA (Y21W), the BLUF-containing activation of photopigment and pucA photoreceptor from *Rhodobacter sphaeroides*, results in the formation of the anion flavin radical, compared with the neutral flavin radical formed in the WT (Lukacs *et al.*, 2014 ▸). In PixD, the BLUF protein from *Synechocystis*, the equivalent mutation (Y8W) blocks the formation of the signalling state (Bonetti *et al.*, 2009 ▸), whereas in OaPAC_BLUF_ a Y6W mutation was recently introduced to dissect the forward electron and proton transfer following blue-light excitation of the flavin (Chen *et al.*, 2023 ▸). To investigate the structural role of this residue, we have replaced Tyr6 by a tryptophan (Y6W), a mutation which is expected to disturb the hydrogen-bond network [Fig. 1 ▸(*b*)] around the flavin and at the same time form a photoinactive enzyme (Lukacs *et al.*, unpublished results). The Y6W mutation does not result in a change of the space group (which remains *P*2_1_2_1_2) or unit-cell parameters (Table 1 ▸), but it seems to induce the anticipated change in the hydrogen-bond network around the flavin. In particular, loss of the hydrogen bond between Tyr6 and Gln48 due to the Y6W mutation results in changes of up to 0.3 Å in the hydrogen-bond distances of several residues (Asn30, Met92, Arg63 and Asp67) in the flavin environment (Table 3 ▸). The side chain of Lys29 also adopts a different orientation, pointing away from the flavin in the Y6W mutant [Fig. 5 ▸(*b*)] compared with WT OaPAC [Fig. 5 ▸(*c*)]. However, the most extensive change is the ∼85° rotation around the CG—CD bond of the Gln48 residue, with the distances of the amide nitrogen and carbonyl oxygen of the side chain of Gln48 to the N5 of the flavin changing from 3.3 and 3.1 Å to 3.1 and 4.0 Å, respectively. The isoalloxazine moiety also rotates a few degrees in the Y6W mutant and displays an average shift of 0.2 Å, and the side chain of Met92 rotates ∼20°, whereas no notable changes are observed for Trp90, which retains a Trp_out_ orientation pointing away from the flavin as observed in the WT [Fig. 5 ▸(*c*)] and previously published WT OaPAC structures (Ohki *et al.*, 2016 ▸, 2017 ▸; Chretien *et al.*, 2024 ▸). The positions of the rest of the nearby residues also do not change in the Y6W mutant [Fig. 5 ▸(*a*)]. All distances are summarized in Table 3 ▸ and Supplementary Fig. S5. It should be pointed out that based on spectroscopic and computational studies for AppA and PixD (Tolentino Collado *et al.*, 2024 ▸; Lukacs *et al.*, 2022 ▸; Hontani *et al.*, 2023 ▸; Hashem *et al.*, 2023 ▸), a 180° rotation of the side chain of the glutamine residue and a keto–enol tautomerism of the glutamine residue have been proposed to take place. These changes in the side chain of the glutamine residue are both triggered by the electron and proton transfer from the tyrosine residue to the oxidized flavin and are considered to be responsible for the hydrogen-bond rearrangement around the flavin in the WT protein upon blue-light excitation. In the recent TR-SFX study of ATP-bound OaPAC, a >180° rotation of the side chain of Gln48 was observed within a few microseconds after blue-light excitation, followed by a methionine/tryptophan switch (Chretien *et al.*, 2024 ▸). The latter residues have received special attention, since different conformations have been reported, as discussed below, and have been considered to be responsible for signal transduction in the BLUF domains as summarized in Karadi *et al.* (2020 ▸). In particular, in AppA the nearby methionine on the β5 strand of the BLUF domain has been shown to exist in two conformations, Met_in_ and Met_out_ (Jung *et al.*, 2006 ▸). Our studies of AppA_BLUF_ have revealed a dynamic picture for the tryptophan residue Trp104, with the dominant population pointing away from the flavin in the dark-adapted state (17.7–20.5 Å) and moving closer to the flavin in the light-adapted state (8.3–9.5 Å) (Karadi *et al.*, 2020 ▸). In contrast, recent solution ^19^F-NMR studies on AppA_BLUF_ have pointed towards a tryptophan (Trp104) that is entirely buried in the protein scaffold and a predominant Trp_in_ configuration in OaPAC_BLUF_ (Zhou *et al.*, 2024 ▸). Recent molecular-dynamics simulations on the microsecond timescale in AppA_BLUF_ have revealed an overall change in the structure of the BLUF domain, with a sliding of the flavin ring within the pocket towards His85, cleavage of the of Asp82–Agr84 salt bridge with the subsequent formation of a new salt bridge between Arg84 and Asp28, an increase of the angle formed by the two α-helices, and structural changes in the β-sheet originating from Gln63 and/or Arg84 (Hashem *et al.*, 2023 ▸).

**Figure 6:**
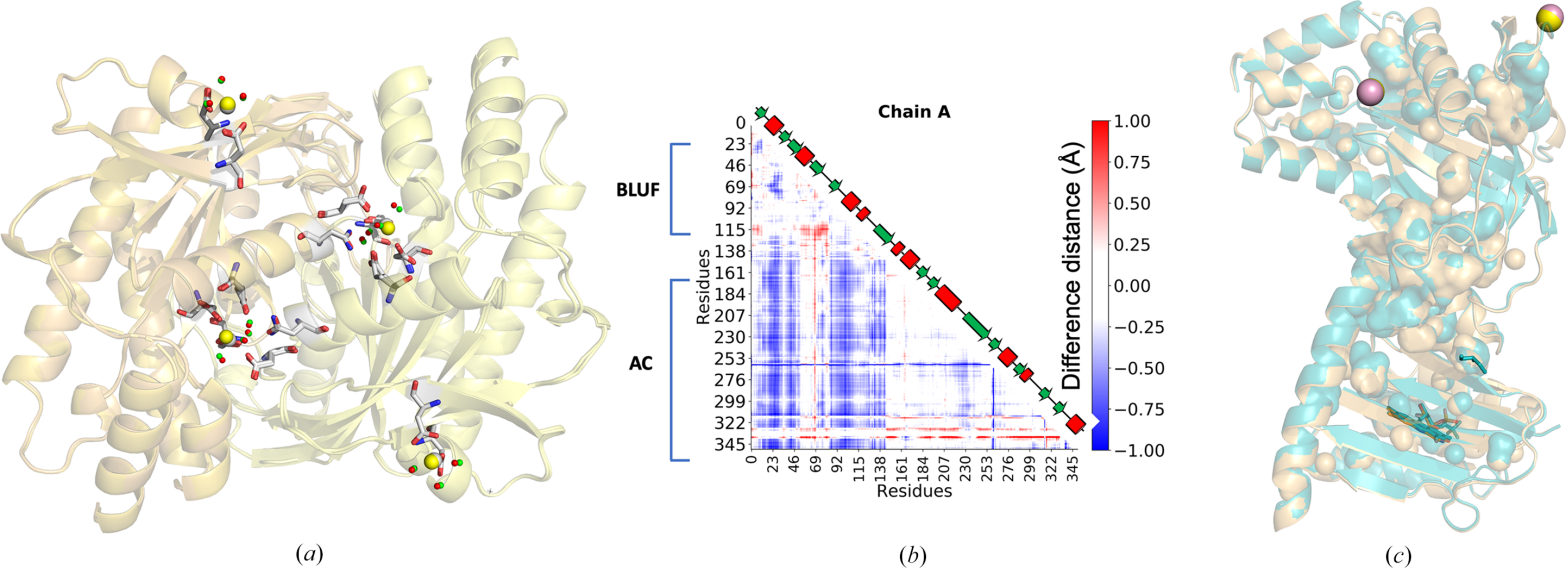
(*a*) Superimposition of the AC domain of the WT OaPAC cryo-MX structure (PDB entry 9f1y) and the Y6W OaPAC cryo-structure (PDB entry 9f1z) showing amino-acid residues coordinated to the calcium ions (WT, grey spheres; Y6W, yellow spheres) and water molecules (WT, pink spheres; Y6W, green spheres) coordinated to calcium ions. (*b*) DDM calculated between the cryo-MX synchrotron structure of WT OaPAC and Y6W OaPAC. Red and blue indicate increasing and decreasing distance with respect to the cryo-structure of WT OaPAC, respectively. (*c*) Superimposition of monomeric OaPAC (WT, orange; Y6W, cyan; calcium ions in WT, pink spheres; calcium ions in Y6W, yellow spheres) showing cavities calculated with *PyMOL*.

In the AC domains of both the WT and Y6W cryo-MX synchrotron structures, there are two types of calcium binding sites (Supplementary Figs. S6 and S7) as observed in our WT OaPAC SFX structure (Fig. 3 ▸). At the internal calcium binding sites, calcium is coordinated by four water molecules in both structures and by five amino-acid residues (Asp156, Asp200, Asp270, Asn273 and Glu279) [Supplementary Figs. S6(*d*), S6(*e*), S7(*b*) and S7(*c*)]. Calcium also binds at the surface of the protein, forming bonds to three water molecules and two amino-acid residues (Asp310 and Asp321) [Supplementary Figs. S6(*b*), S6(*c*), S7(*d*) and S7(*e*)]. Inspection of the AC domains [Fig. 6 ▸(*a*)] reveals a significant change in the accessible solvent area (ASA) in the Y6W mutant (277 Å^2^) compared with the WT (315 Å^2^) (Supplementary Fig. S8), suggesting that a change in the hydrogen-bond environment of the flavin may affect the conformation of the AC domains. The DDM [Fig. 6 ▸(*b*)] also shows changes in the distances in the AC domain. In particular, in the Y6W OaPAC mutant the BLUF domain is closer to the AC domain compared with the analogous distance in WT OaPAC. Small differences are also observed in their internal cavities, as illustrated in Fig. 6 ▸(*c*), whereas the theoretical SAXS parameters show small differences between the two structures in the *R*_g_ and *D*_max_ values, Kratky plot and pair distribution function [Fig. 2 ▸(*b*), Supplementary Fig. S3].

### 3.6. Effect of the crystallization conditions on the crystal structure of WT OaPAC

**Figure 7:**
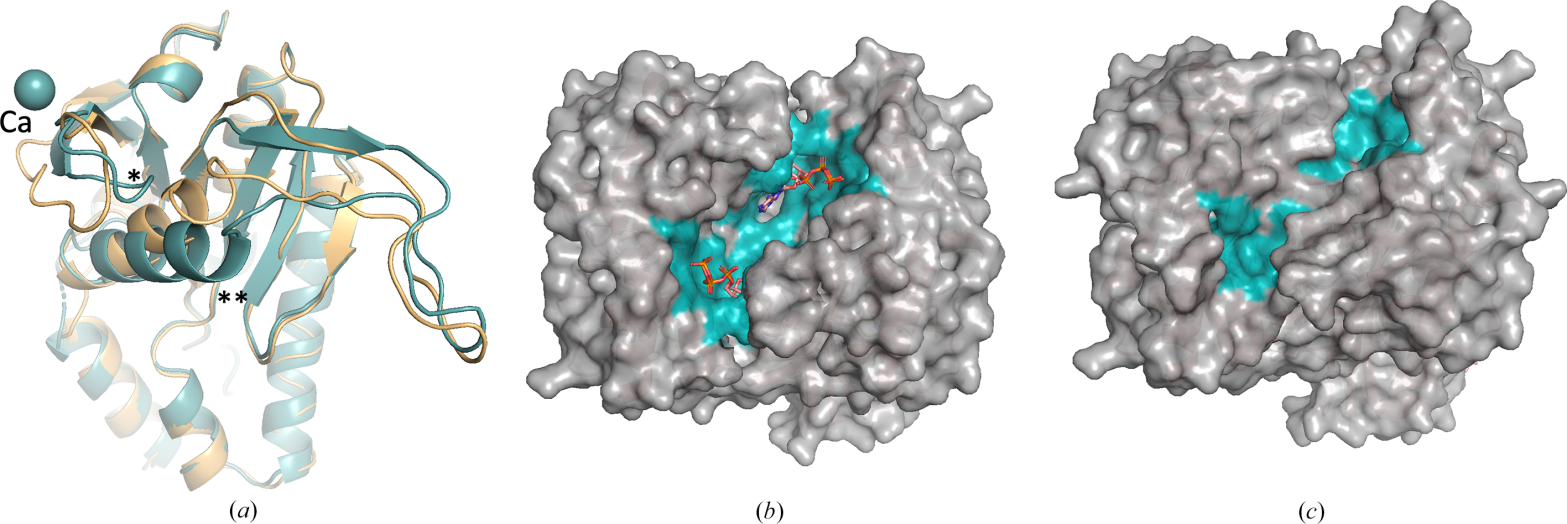
(*a*) Comparison of our substrate-free WT OaPAC cryo-MX synchrotron structure (green; PDB entry 9f1y) with the ATP-bound OaPAC SFX structure (PDB entry 8qfh, yellowish-orange) showing the loop (labelled with an asterisk) that pushes down a helix (labelled with two asterisks). (*b*) ATP binding pocket in the ATP-bound OaPAC SFX structure of Chretien and coworkers (PDB entry 8qfh). (*c*) ATP binding pocket in our substrate-free WT OaPAC cryo-MX synchrotron structure (PDB entry 9f1y).

Comparison of our OaPAC structures with the recently released OaPAC structures determined by Chretien *et al.* (2024 ▸) and the available OaPAC structures of Ohki *et al.* (2016 ▸, 2017 ▸) reveals some interesting features that presumably arise from the different crystallization conditions used [0.06 *M* divalents, 0.1 *M* Tris–Bicine pH 8.5, 30%(*v*/*v*) PEG 550 MME/PEG 20 000 (this work), 100 m*M* SPG buffer pH 7.0, 1.2 *M* disodium succinate, 100 m*M* guanidine–HCl, 5 m*M* MgCl_2_ (Chretien *et al.*, 2024 ▸) and 0.1 *M* sodium citrate pH 5.0, 10% PEG 20 000, 5 m*M* magnesium chloride, 5 m*M* ApCpp (Ohki *et al.*, 2016 ▸)]. As discussed in Section 3.1, OaPAC has been crystallized in various crystal forms: *C*222_1_ (PDB entries 8qfe, 8qff and 8qfh), *P*2_1_2_1_2_1_ (PDB entry 4yut) and *P*6_1_22 (PDB entry 4yus) (Table 2 ▸). In our preparation, OaPAC crystallized in space group *P*2_1_2_1_2. Fig. 7 ▸, which compares the AC domains of our cryo-MX synchrotron OaPAC structure with the SFX ATP-bound OaPAC structure determined by Chretien and coworkers, reveals a significant reorientation of a helix in the ATP-binding domain in our conditions [Fig. 7 ▸(*a*)]. This reorientation seems to originate from a loop placed on top of the α5 helix, which in our preparation seems to push down the α5 helix, resulting in the observed conformation of the helix. As a result, the ASA for the ATP pocket, as calculated using the *PISA* server (https://www.ebi.ac.uk/pdbe/pisa/) and a custom-built Python script, is reduced from 511 Å^2^ in the cryo-structure of Chretien and coworkers (PDB entry 8qfe) [Supplementary Fig. S7(*a*)] to 315 Å^2^ in our cryo-structure [Fig. 7 ▸(*c*) and Supplementary Fig. S7(*d*)]. This is a significant reduction, which results in a close conformation that may prevent ATP from binding. Notably, ATP can bind to OaPAC crystallized in conditions supporting an open conformation (RT, ATP-bound OaPAC; PDB entry 8qfh) [Fig. 7 ▸(*b*) and Supplementary Fig. S7(*e*)]. Similar to the structures of Chretien *et al.* (2024 ▸), those reported by Ohki *et al.* (2016 ▸, 2017 ▸) also display an open conformation for the ATP pocket [Supplementary Figs. S7(*b*) and S7(*c*)] compared with our cryo-structure. These findings highlight the importance of having an open conformation for ATP binding and are in line with a previous study in which the hydrophobic C-terminus of OaPAC was proposed to interact with the ATP binding site or with a site that is crucial for conformational changes in the AC domains, restricting OaPAC from producing cAMP from ATP (Hirano *et al.*, 2019 ▸). A *B*-factor putty representation showing differences in the *B* factors between the available OaPAC structures is shown in Supplementary Fig. S9. These differences reflect the effect that ligand binding and various crystallization conditions and temperatures can have on the structure of OaPAC.

## 4. Conclusions

This study presents, for the first time, RT structures of substrate-free OaPAC obtained at an XFEL and also at a synchrotron. It highlights how the crystallization conditions can influence crystal packing, which may result in conformational differences affecting ligand binding, and how changes in the sensor domain of a protein can affect its effector domain. It also paves the way for time-resolved studies at XFELs (TR-SFX) and synchrotrons (TR-SSX), which will provide structural information on the early intermediates (picosecond–millisecond) forming upon blue-light excitation of substrate- and radiation damage-free OaPAC. These structures will further allow a correlation of the available spectroscopic signatures of the substrate-free enzyme (Tolentino Collado *et al.*, 2022 ▸, 2024 ▸) with the early-formed structural intermediates, making it possible to obtain a complete picture of the photoactivation mechanism of the enzyme. In particular, we will be able to visualize the spectroscopically reported flavin intermediates formed upon blue-light excitation (Tolentino Collado *et al.*, 2022 ▸), together with structural rearrangements of critical residues in the hydrogen-bond network of the flavin taking place on the picosecond timescale. In addition, snapshots of the movement of the β5 strand in the BLUF domain, in the α3 helix and in the AC domains occurring on the millisecond timescale might be obtained. Comparison of the structural intermediates describing the signal transduction pathway between substrate-free and substrate-bound OaPAC may shed light on the dynamic processes of substrate binding to OaPAC that lead to product formation and in general provide insight into the mechanisms of action of light-activated enzymes.

## Supplementary Material

PDB reference: wild-type OaPAC, SFX structure, 9f1w

PDB reference: SSX structure, 9f1x

PDB reference: cryo-MX structure, 9f1y

PDB reference: Y6W OaPAC, cryo-MX structure, 9f1z

Supplementary figures, table and note. DOI: 10.1107/S2052252524010170/car5001sup1.pdf

Video showing jetting of the slurry of OaPAC microcrystals tested at the EuXFEL XBI laboratory. DOI: 10.1107/S2052252524010170/car5001sup2.mp4

Experimental data for cryo-structures of WT OaPAC and Y6W OaPAC: https://doi.org/10.15151/ESRF-DC-1897099364

Data recorded for the experiment at the European XFEL: https://doi.org/10.22003/XFEL.EU-DATA-003018-00

## Figures and Tables

**Table 1:** Data-collection and refinement statistics for WT OaPAC and Y6W OaPAC Statistics for the highest resolution shell and standard deviations of the unit-cell parameters are shown in parentheses.

	WT, SFX	WT, SSX	WT, cryo-MX	Y6W, cryo-MX
PDB entry	9f1w	9f1x	9f1y	9f1z

Data collection				
Data-collection temperature (K)	294 ± 1	293 ± 1	100	100
Space group	*P*2_1_2_1_2	*P*2_1_2_1_2	*P*2_1_2_1_2	*P*2_1_2_1_2
*a* (Å)	102.9 (0.32)	101.5 (0.17)	101.3	100.5
*b* (Å)	54.9 (0.19)	53.9 (0.11)	53.5	52.7
*c* (Å)	73.0 (0.23)	72.05 (0.13)	71.8	71.5
α (°)	90.0 (0.3)	90.0 (0.2)	90.0	90.0
β (°)	90.0 (0.3)	90.0 (0.2)	90.0	90.0
γ (°)	90.0 (0.3)	90.0 (0.2)	90.0	90.0
Resolution (Å)	27–1.75 (1.78–1.75)	59–1.80 (1.86–1.80)	42.89–1.95 (2.02–1.95)	46.66–1.95 (2.02–1.95)
Wilson *B* factor (Å^2^)	39.44	35.69	28.79	35.37
〈*I*/σ(*I*)〉	7.4 (1.1)	7.8 (0.6)	14.5 (1.6)	12.8 (1.1)
*R*_split_ (%)	8.5 (90.8)	7.4 (190.7)	N/A	N/A
*R*_merge_ (%)	N/A	N/A	7.9 (122.5)	5.4 (121.0)
CC_1/2_ (%)	99.1 (58.7)	99.6 (19.1)	99.9 (70.2)	99.60 (48.0)
CC* (%)	99.8 (86.0)	99.9 (56.7)	100.0 (90.8)	99.90 (80.5)
Completeness (%)	100.0 (100.0)	100.0 (100.0)	99.5 (99.8)	98.0 (97.8)
Multiplicity	453 (274)	595 (407)	6.4 (6.5)	3.8 (3.8)
No. of indexed lattices	27202	33847	N/A	N/A
No. of reflections	18173160 (738236)	21976641 (1474529)	186652 (13233)	106179 (7386)
No. of unique reflections	40124 (2694)	36950 (3615)	29029 (2848)	27876 (2727)

Refinement				
*R*_work_ (%)	19.3 (30.8)	18.4 (36.9)	20.1 (31.3)	19.6 (38.9)
*R*_free_ (%)	21.9 (31.0)	21.2 (37.8)	22.7 (32.2)	23.3 (44.8)

No. of atoms				
Total	2924	2960	3004	2991
Protein	2778	2835	2772	2781
Ligand/ion	33	33	33	39
Water	113	92	199	171
R.m.s.d., bond lengths (Å)	0.009	0.008	0.008	0.008
R.m.s.d., angles (°)	0.88	0.97	0.92	1.05
Ramachandran favoured (%)	98.24	98.83	99.13	97.71
Ramachandran allowed (%)	1.76	0.88	0.87	2.29
Ramachandran outliers (%)	0.00	0.29	0.00	0.00
Rotamer outliers (%)	1.90	1.54	0.32	2.29
Clashscore	5.32	5.85	4.26	7.59
Average *B* factors (Å^2^)				
Overall	46.63	44.25	39.49	46.67
Macromolecules	46.27	44.07	39.25	46.09
Ligands	53.71	56.03	48.03	52.99
Solvent	53.44	45.71	41.52	54.73

**Table 2:** A summary of all available OaPAC structures in the dark-adapted state containing FMN as the chromophore

Protein	PDB code	Resolution (Å)	Space group	*a*, *b*, *c* (Å); α, β, γ (°)	Structure	Temperature	Reference
OaPAC	4yut	2.9	*P*2_1_2_1_2_1_	85.3, 100.7, 120.7; 90, 90, 90	WT, cryo-MX, synchrotron	100 K	Ohki *et al.* (2016 ▸)
OaPAC/ApCpp added	4yus	1.8	*P*6_1_22	76.8, 76.8, 204.8; 90, 90, 120	WT, cryo-MX, synchrotron	100 K	Ohki *et al.* (2017 ▸)
OaPAC	8qfe	1.5	*C*222_1_	52.9, 146.2, 103.6; 90, 90, 90	WT, cryo-MX, synchrotron	100 K	Chretien *et al.* (2024 ▸)
OaPAC/ATP	8qff	2.1	*C*222_1_	54.4, 146.4, 104.9; 90, 90, 90	WT, cryo-MX, synchrotron	100 K	Chretien *et al.* (2024 ▸)
OaPAC/ATP	8qfh	1.8	*C*222_1_	54.3, 145.8, 105.3; 90, 90, 90	WT, SFX	RT	Chretien *et al.* (2024 ▸)
OaPAC	9f1w	1.75	*P*2_1_2_1_2	102.9, 54.9, 72.98; 90, 90, 90	WT, SFX	RT	This work
OaPAC	9f1x	1.8	*P*2_1_2_1_2	101.5, 53.9, 72.05; 90, 90, 90	WT, SSX	RT	This work
OaPAC	9f1y	1.95	*P*2_1_2_1_2	101.3, 53.5, 71.8; 90, 90, 90	WT, cryo-MX, synchrotron	100 K	This work
Y6W OaPAC	9f1z	1.95	*P*2_1_2_1_2	100.5, 52.7, 71.5; 90, 90, 90	Y6W, cryo-MX, synchrotron	100 K	This work

**Table 3:** Comparison of (hydrogen-bond) distances of the side chains of residues around the flavin in WT OaPAC and Y6W OaPAC

(Hydrogen-bond) distance (Å)	WT OaPAC (PDB entry 9f1y)	Y6W OaPAC (PDB entry 9f1z)
Lys29–PO4(FMN)	4.1	4.6
Lys29–C2=O(FMN)	5.3	7.4
Gln48(N)–N5(FMN)	3.3	3.1
Gln48(O)–N5(FMN)	3.1	4.0
Met92(S)–Gln48(N)	3.4	3.8
Gln48(O)–Tyr6(O)/Trp6(N)	2.5	3.0
Asn30(O)–N3(FMN)	2.9	3.6
Asn30(N)–C4=O(FMN)	3.1	3.1
Arg63(NH2)–C2=O(FMN)	3.1 and 4.6	2.9 and 4.7
Asp67–O(C3′-FMN)	3.2	3.2
Asp67–O(C2′-FMN)	3.0	2.8
