# Supplementary material for: Crystal structure of a bacterial photoactivated adenylate cyclase determined by serial femtosecond and serial synchrotron crystallography
Source: IUCrJ. 2024 Oct 29;11(Pt 6):991–1006. doi: 10.1107/S2052252524010170 (PMC11533990; doi:10.1107/S2052252524010170)
Supplement: Supplementary file 1 [file m-11-00991-sup1.pdf]

IUCrJ

Volume 12 (2025)

Supporting information for article:

Crystal structure of a bacterial photoactivated adenylate cyclase determined at room temperature by serial femtosecond crystallography

Sofia M. Kapetanaki, Nicolas Coquelle, David von Stetten, Martin Byrdin, Ronald Rios-Santacruz, Richard Bean, Johan Bielecki, Mohamed Boudjelida, Zsuzsana Fekete, Geoffrey W. Grime, Huijong Han, Caitlin Hatton, Sravya Kantamneni, Konstantin Kharitonov, Chan Kim, Marco Kloos, Faisal H. M. Koua, Iñaki de Diego Martinez, Diogo Melo, Lukas Rane, Adam Round, Ekaterina Round, Abhisakh Sarma, Robin Schubert, Joachim Schulz, Marcin Sikorski, Mohammad Vakili, Joana Valerio, Jovana Vitas, Raphael de Wijn, Agnieszka Wrona, Ninon Zala, Arwen Pearson, Katerina Dörner, Giorgio Schirò, Elspeth F. Garman, András Lukács and Martin Weik

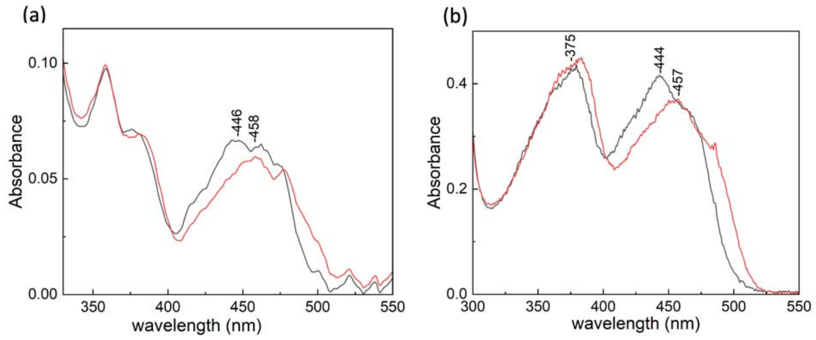

Figure S1 . UV-vis spectra of OaPAC in the dark- (black line) and light-adapted state (red line) (a) in the crystalline form and in (b) solution.

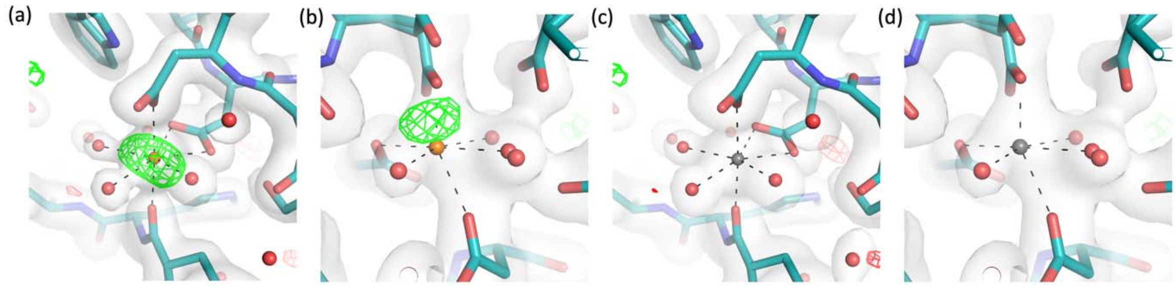

Figure S2 Ion modelling in WT OaPAC crystal structures. Based on crystallisation conditions, either magnesium or calcium ions could be modelled at specific sites in the protein. Two models were generated (one with each type of ions) and refined against the EuXFEL RT diffraction data. Resulting signal in the mFo-DFc maps is displayed as green (contoured at +3.5 σ) and red (contoured at -3.5 σ) meshes. Panels (a) and (b) represent the modelling of Mg^{2+} ion (orange spheres) in the two different regions. The protein model is represented as teal sticks, and water molecules as red spheres. The 2mFo-DFc map is represented as an isosurface contoured at 1.5 σ. The green density indicates a lack of electrons in the model. Panels (c) and (d) represent models with calcium ions (grey sphere), but with no idication of a signal in the mFo-DFc map, thereby favouring the modelling of calcium ions in the structures.

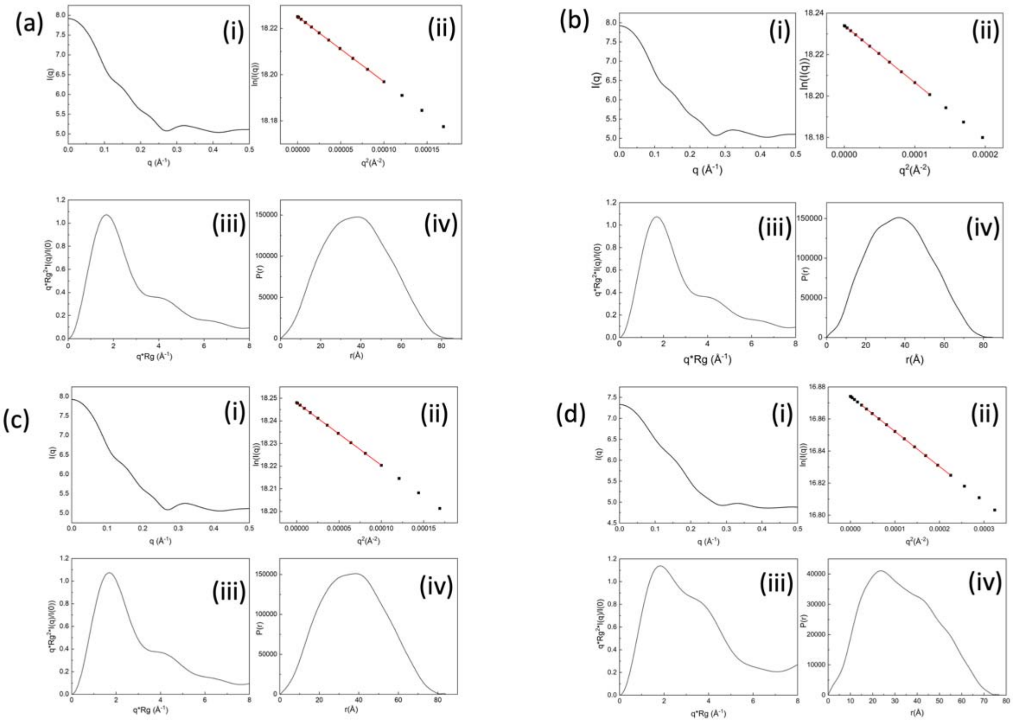

Figure S3 Theoretical SAXS data derived from the available crystal structures of OaPAC using the FoXS server. (a) WT OaPAC SFX structure (pdb 9f1w), (b) WT OaPAC SSX structure (pdb 9f1x), (c) WT OaPAC cryo-MX synchrotron structure (pdb 9f1y), (d) Y6W OaPAC cryo-MX synchrotron structure (pdb 9f1z). (i) theoretical SAXS profile (ii) Guinier analysis (iii) Kratky plot (iv) Pair distribution function, P(r).

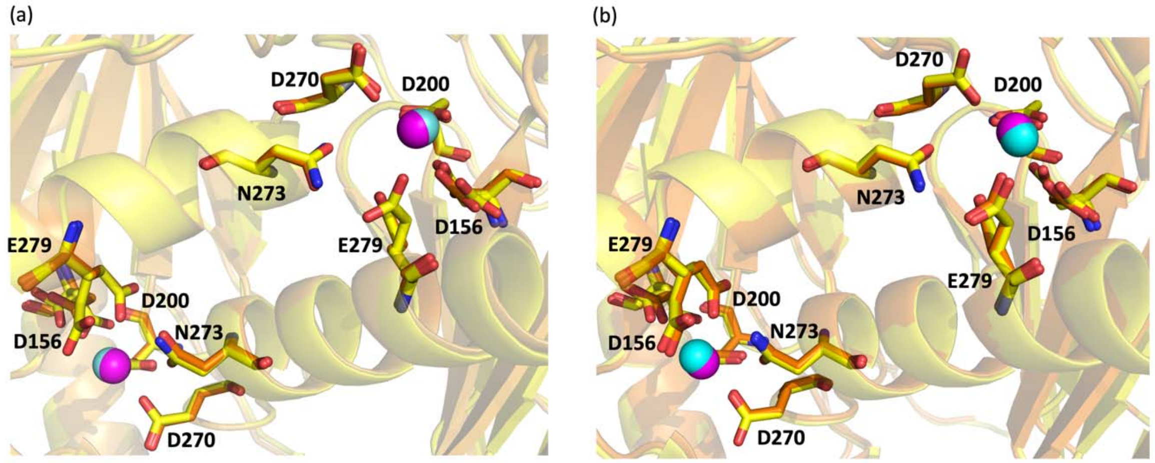

Figure S4 (a) Superimposition and zoom-in in the internal calcium binding sites of the AC domains of the WT OaPAC SFX structure (yellow) (pdb 9f1w) and of the WT OaPAC cryo-MX synchrotron structure (orange) (pdb 9f1y) showing amino acid residues coordinated to the calcium ions (SFX; pink spheres, cryo; cyan spheres) (b) Superimposition of the AC domains of the WT OaPAC SFX structure (yellow) and of the WT OaPAC SSX structure (orange) showing amino acid residues coordinated to the calcium ions (SFX; pink spheres, SSX; cyan spheres).

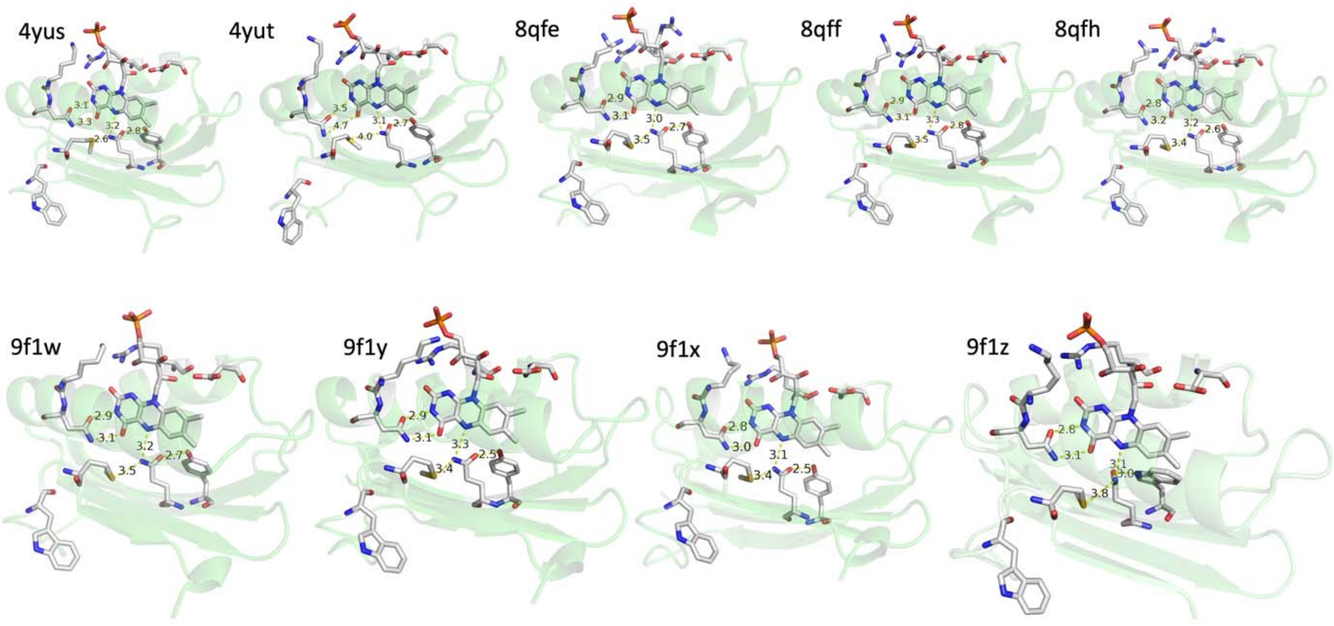

Figure S5 Flavin active site of OaPAC showing key amino acid residues participating in the hydrogen bond network and corresponding distances.

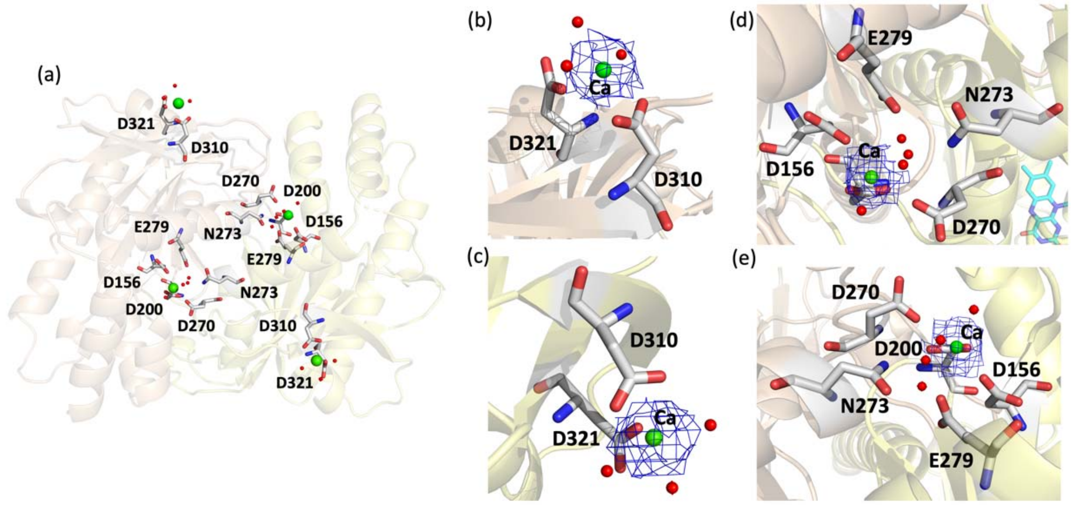

Figure S6 (a) AC domain of WT OaPAC cryo-MX synchrotron structure (pdb 9f1y) showing amino acid residues coordinated to the calcium ions (green spheres). Water molecules coordinated to the calcium ions are shown as red spheres. (b) and (c) zoom-in on the calcium binding sites on the surface of WT OaPAC (d) and (e) zoom-in on the dimer interface and the calcium binding sites of WT OaPAC; the 2mFo-DFc map (contoured at +1.0 σ) is coloured in blue and shown only for the calcium ions.

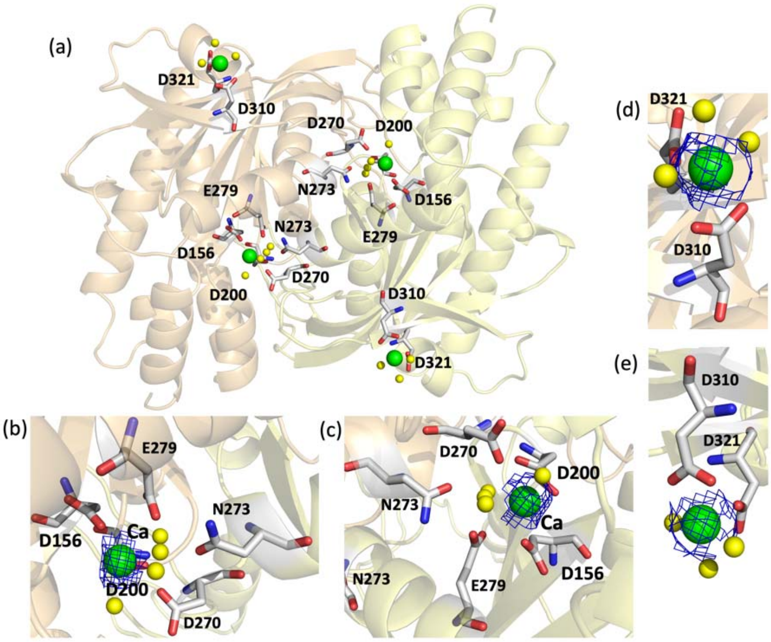

Figure S7 (a) AC domain of Y6W OaPAC cryo-MX synchrotron structure, (pdb 9f1z) showing amino acid residues coordinated to the calcium ions (green spheres). Water molecules coordinated to the calcium ions are shown as yellow spheres. (b) and (c) zoom-in on the dimer interface and the calcium binding sites of Y6W OaPAC. d) and e) zoom-in on the calcium binding sites on the surface of Y6W OaPAC (d) and (e) the 2mFo-DFc map (contoured at +1.0 σ) is coloured in blue and shown only for the calcium ions (green spheres; water molecules: yellow spheres).

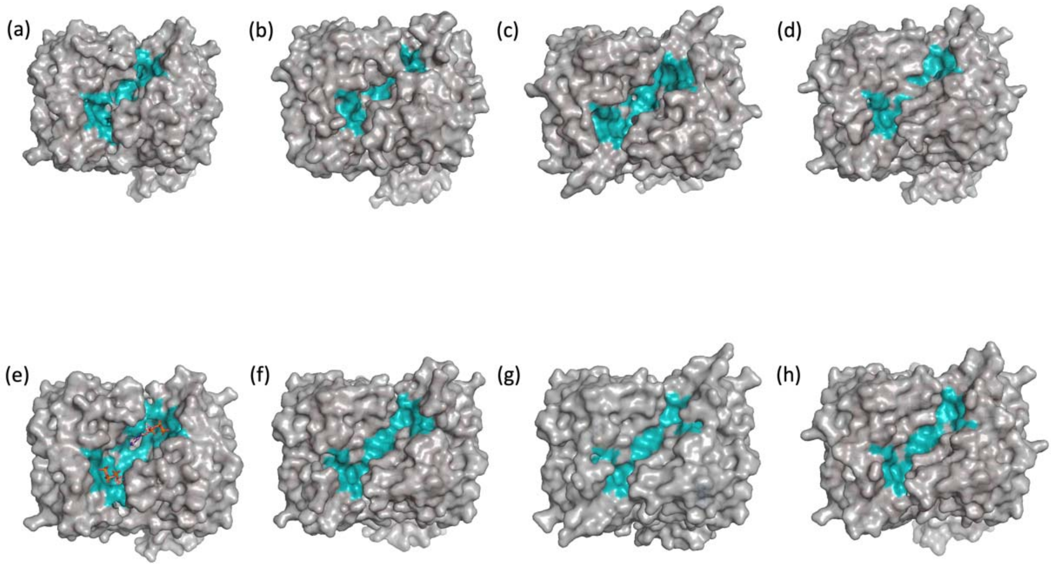

Figure S8 Comparison of the ATP binding pocket shown for the (a) cryo-MX ATP-free OaPAC structure of Chretien *et al*. (pdb 8qfe) (511 Å^2), (b) WT OaPAC cryo-MX synchrotron structure (pdb 4yut) (328 Å^2), (c) WT OaPAC cryo-MX synchrotron structure (pdb 4yus) (500 Å^2), (d) our WT OaPAC cryo-MX synchrotron structure (pdb 9f1y) (315 Å), (e) ATP-bound WT OaPAC SFX structure of Chretien *et al.* (pdb 8qfh) (663 Å^2), (f) our WT OaPAC SFX structure (pdb 9f1w) (378 Å^2), (g) our WT OaPAC SSX structure (pdb 9f1x) (269 Å^2) and (h) our Y6W OaPAC cryo-MX synchrotron structure (pdb 9f1z) (277 Å^2). The accessible solvent area (ASA) for the ATP pocket is given in parentheses.

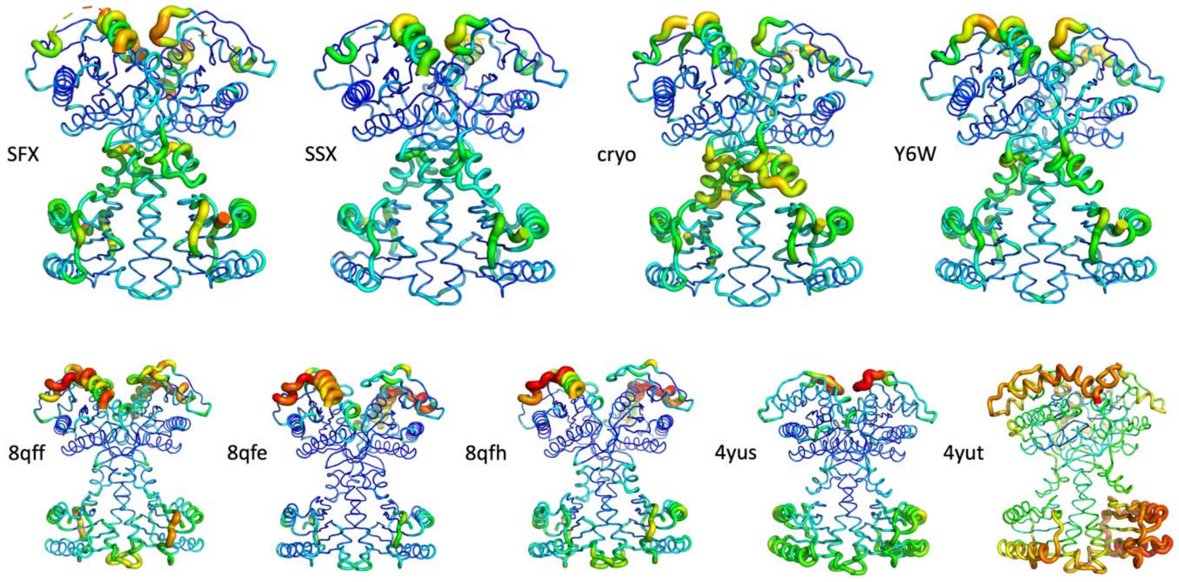

Figure S9 Comparison of the *B*-factor putty representations for all OaPAC structures. Red colours and a wider tube indicate regions with higher B-factors, whereas shades of blue and a narrower tube indicate regions with lower B-factors.

Table S1 Secondary structure elements extracted from the pdb files.

WT OaPAC structure	helix (%)	sheet (%)	turn (%)	coil (%)
SFX (9f1w)	42.0 (0.8*)	29.6 (1.9*)	7.5 (1.5*)	20.9 (1.2*)
SSX (9f1x)	41.9 (0.7*)	29.5 (1.8*)	7.5 (1.5*)	21.1 (1*)
cryo-MX (9f1y)	42.9	29.1	8.9	19.1
cryo-MX (4yut)	41.0	26.1	7.7	25.2
cryo-MX (8qfe)	39.7	28.0	10.3	22.0
Mean ± SD 4yut/9f1y/8qfe	41.2 ± 1.6	27.7 ± 1.5	9 ± 1.3	22.1 ± 3.05

*Difference between values of the RT structures (SFX, SSX) and mean values of the cryo-structures (4yut, 9f1y, 8qfe) of OaPAC. The values of the standard deviation and the difference between RT values and the mean values of the cryo-structures are comparable, showing that the difference in the secondary structure elements is not significant.

Note S1. Residues 332-337 are not resolved in our SFX structure. In the structures of Chretien *et al.* they are part of a coil, in the structures of Ohki *et al.* they are part of a helix, whereas in our cryo- structures are resolved and they are part of a coil. They are found at the surface of the AC domains and therefore we do not consider them critical for TR-studies to understand the signal transduction mechanism since they are not found in the BLUF domain, in the coil-coil interface or close to the ATP binding site.
